# The gold-ringed octopus (*Amphioctopus fangsiao*) genome and cerebral single-nucleus transcriptomes provide insights into the evolution of karyotype and neural novelties

**DOI:** 10.1186/s12915-022-01500-2

**Published:** 2022-12-27

**Authors:** Dianhang Jiang, Qun Liu, Jin Sun, Shikai Liu, Guangyi Fan, Lihua Wang, Yaolei Zhang, Inge Seim, Shucai An, Xin Liu, Qi Li, Xiaodong Zheng

**Affiliations:** 1 Key Laboratory of Mariculture, Ministry of Education, Ocean University of China, Ocean University of China, Qingdao, 266003 China; 2 Institute of Evolution & Marine Biodiversity (IEMB), Qingdao, 266003 China; 3 BGI-QingDao, BGI-Shenzhen, Qingdao, 266555 China; 4 State Key Laboratory of Agricultural Genomics, BGI-Shenzhen, Shenzhen, 518083 China; 5 Integrative Biology Laboratory, College of Life Sciences, Nanjing Normal University, Nanjing, 210046 China; 6 School of Biology and Environmental Science, Queensland University of Technology, Brisbane, 4000 Australia; 7 The Affiliated Hospital of Qingdao University, Qingdao, China

**Keywords:** Cephalopod, Octopoda, Chromosome, Genome, Single cell, Supra-esophageal brain

## Abstract

**Background:**

Coleoid cephalopods have distinctive neural and morphological characteristics compared to other invertebrates. Early studies reported massive genomic rearrangements occurred before the split of octopus and squid lineages (Proc Natl Acad Sci U S A 116:3030-5, 2019), which might be related to the neural innovations of their brain, yet the details remain elusive. Here we combine genomic and single-nucleus transcriptome analyses to investigate the octopod chromosome evolution and cerebral characteristics.

**Results:**

We present a chromosome-level genome assembly of a gold-ringed octopus, *Amphioctopus fangsiao*, and a single-nucleus transcriptome of its supra-esophageal brain. Chromosome-level synteny analyses estimate that the chromosomes of the ancestral octopods experienced multiple chromosome fission/fusion and loss/gain events by comparing with the nautilus genome as outgroup, and that a conserved genome organization was detected during the evolutionary process from the last common octopod ancestor to their descendants. Besides, protocadherin, GPCR, and C2H2 ZNF genes are thought to be highly related to the neural innovations in cephalopods (Nature 524:220–4, 2015), and the chromosome analyses pinpointed several collinear modes of these genes on the octopod chromosomes, such as the collinearity between PCDH and C2H2 ZNF, as well as between GPCR and C2H2 ZNF. Phylogenetic analyses show that the expansion of the octopod protocadherin genes is driven by a tandem-duplication mechanism on one single chromosome, including two separate expansions at 65 million years ago (Ma) and 8–14 Ma, respectively. Furthermore, we identify eight cell types (i.e., cholinergic and glutamatergic neurons) in the supra-esophageal brain of *A. fangsiao*, and the single-cell expression analyses reveal the co-expression of protocadherin and GPCR in specific neural cells, which may contribute to the neural development and signal transductions in the octopod brain.

**Conclusions:**

The octopod genome analyses reveal the dynamic evolutionary history of octopod chromosomes and neural-related gene families. The single-nucleus transcriptomes of the supra-esophageal brain indicate their cellular heterogeneities and functional interactions with other tissues (i.e., gill), which provides a foundation for further octopod cerebral studies.

**Supplementary Information:**

The online version contains supplementary material available at 10.1186/s12915-022-01500-2.

## Background

Extant cephalopods can be divided into two major clades, Coleoidea and Nautiloidea. Coleoid cephalopods (octopuses, cuttlefish, and squids) have a complex nervous system that stands out in invertebrates, which can even rival some vertebrates in neural size and complexity [1]. The nautilus genome experienced slow evolution rates in the coding and non-coding regions and less intron gains/losses than other coleoids [2] and also has slow growth rates in the wild [3]. Considering its phylogenetic position, sister to all the other extant cephalopods, and its slow rate of evolution, nautilus might maintain the plesiomorphic (or less derived) characteristic of the group [4, 5], closely reflecting their ancestral condition compared to its closest relatives. The nautilus has relatively simpler nervous system compared with coleoid cephalopods [5], and this raises an important evolutionary question on how the coleoid neural system evolved.

To address this, coleoid cephalopod genomes are important as they provide essential genetic information that controls individual development and evolution. *Octopus bimaculoides* is the first coleoid cephalopod to be sequenced, followed by four additional octopuses [6–9], two squids [10, 11], and one nautilus [2, 12]. Genomic analyses have revealed that the expansion of a number of gene families (i.e., protocadherins, PCDHs; C2H2 zinc-finger transcription factors, C2H2 ZNF; and G protein-coupled receptors, GPCRs) and chromosome rearrangements are highly related to the neural novelties of coleoid cephalopods [13]. However, it is not clear about the genomic features of these lineage or tissue-specific gene novelties at chromosome level, for instance how they evolved with the genomic organization. Thus, the chromosome-level genome analyses may deepen the understanding of the genome evolution of coleoid cephalopods.

Besides, understanding the cellular heterogeneity of the neural system of coleoid cephalopod is also key to investigate their neural innovations. The supra-esophageal brain (sup-brain) of coleoid cephalopods is structurally the supra-esophageal mass and is the neural center for learning and memory, which is a lineage-specific innovation in Mollusca [14–16] and is analogous to the cerebral structures in vertebrates [17]. Previous studies have focused on the development, neuroanatomy, and neurobiology of this organ at morphological level [18–23]. These studies have provided fundamental insights into how the supra-esophageal brain is organized, yet how they function relative to multiple behaviors (i.e., learning, task solving, and memory) is still obscure. The cellular composition, sub-functionalization, and molecular evolution of the supra-esophageal brain remain essential to be addressed and can be likely revealed by single-cell analyses.

To better understand octopod evolution and neural novelties, we sequenced a chromosomal-level genome of a gold-ringed octopus, *A. fangsiao*, and a single-nucleus transcriptome of its supra-esophageal brain. We performed chromosome-level synteny analyses to investigate how octopod chromosomes evolved from ancestral cephalopods, and single-nucleus transcriptome analyses to characterize the cellular signatures in octopod sup-brain.

## Results

### Genome sequencing and assembly

The genome of golden-ringed octopus, *A. fangsiao*, was sequenced using Oxford Nanopore Technology (ONT), and a total of 304.9 Gb of clean reads with an average genome coverage of 70.2× and read N50 of 22.96 Kb were produced (Additional file 1: Table s1). The genome assembly is 4.34 Gb in length with a contig N50 size of 2.34 Mb (Additional file 1: Table s2), the assembly quality of which is comparable to or better than those of other available octopod genomes (Additional file 1: Table s3). The high mapping rates of short paired-end DNA (99.33%) and RNA-seq reads from 21 tissues (average 81.53%) indicate that the genome assembly is nearly complete (Additional file 1: Table s2). The genome heterozygosity of *A. fangsiao* was estimated to be 0.96%, which is similar to that of *O. sinensis* (1.10%) [6] and *Hapalochlaena maculosa* (0.95%) [9], but higher than that of *O. bimaculoides* (0.08%) [13]. We anchored 2720 contigs (covering 93.9% of the genome assembly) on 30 linkage groups, which show a high-density genetic linkage map (Additional file 2: Fig. s1). The 30 linkage groups are supported by the karyotype of *A. fangsiao* estimated by the conventional physical method [24].

The *A. fangsiao* genome has 19,654 protein-coding genes; 96.2% of which encode proteins over 100 amino acids (Additional file 1: Table s4). Functional analyses annotated 88.4% of the predicted genes with various databases (see the “Methods” section). The *A. fangsiao* genome contains 2.99 Gb of repeat sequences (covering 68.95% of the genome assembly) (Additional file 1: Table s5), which is by far the largest among the available coleoid cephalopod genomes (37.09%–68.95%) (Additional file 1: Table s6). The high repeat proportion is likely owing to the long-read sequencing of ONT that could jump over highly repetitive regions.

### Phylogenetic analyses and chromosome evolution

**Fig. 1:**
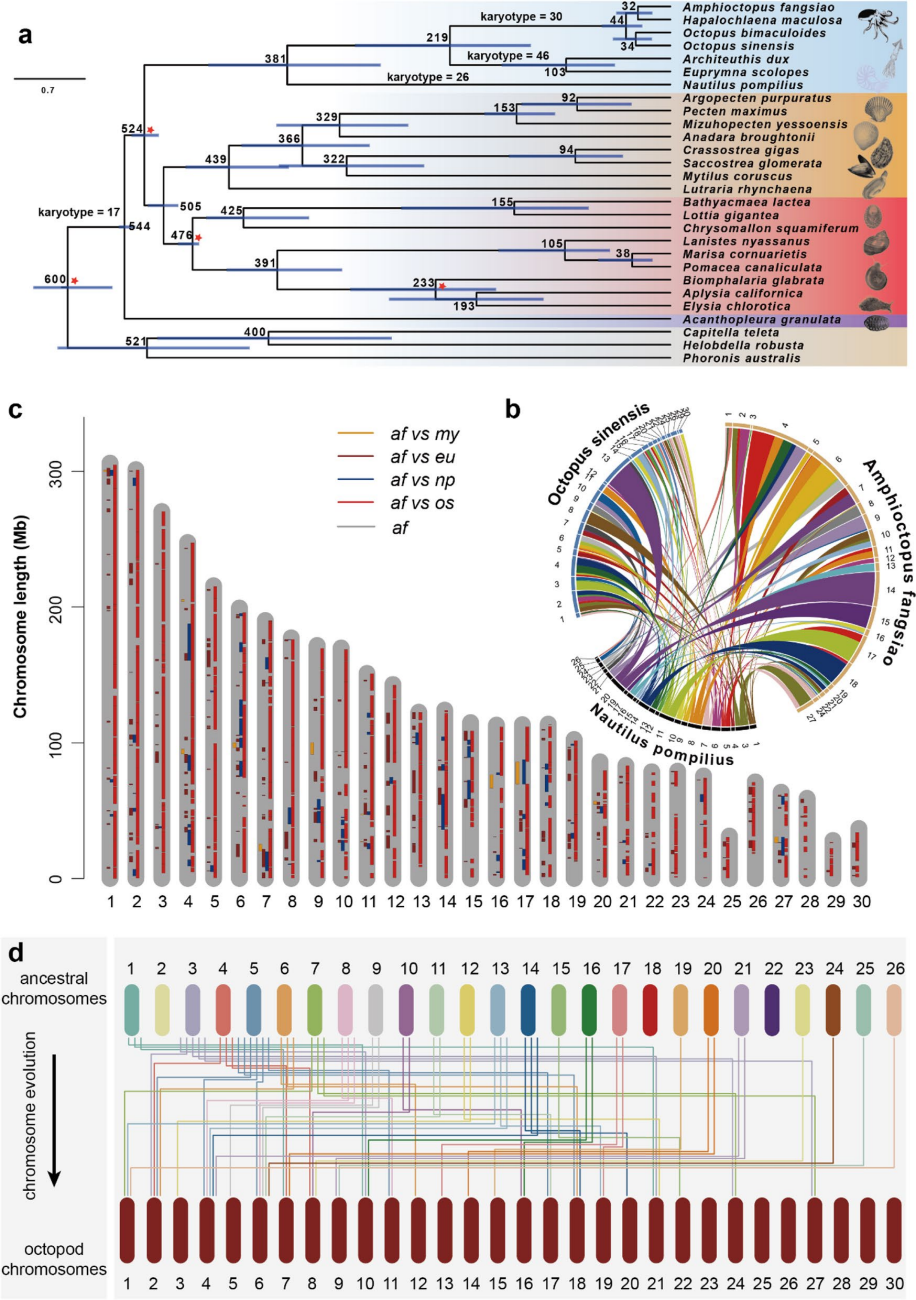
Schematic illustration of the octopod chromosome evolution. **a** Maximum-likelihood (ML) tree of 28 genomes showing the karyotype evolution of cephalopods and divergence times among molluscan lineages. Error bars (blue bar) at nodes indicate 95% confidence levels. The Cephalopoda is highlighted in light blue, Bivalvia in orange, Gastropoda in red, and Polyplacophora in pink. Karyotype data are derived from previous publications [24, 27]. The information of the calibration points used for divergence time estimation was marked as red star at the nodes (details see the “Methods” section). The corresponding ML tree is listed in Fig. S2. **b** Circular plot of the chromosome synteny analyses among *N. pompilius*, *O. sinensis*, and *A. fangsiao*. The inner colored blocks represent the synteny blocks between *N. pompilius* and *A. fangsiao* (or *O. sinensis*), which is used for illustration of the number and length of chromosome synteny blocks, without the chromosome location meaning. The outer segments and numbers represent chromosomes in each species. **c** Schematic illustration of chromosomal synteny blocks between *A. fangsiao* (*af*, gray) and *O. sinensis* (*os*, red), *E. scolopes* (*es*, brown), *N. pompilius* (*np*, blue), or *M. yessoensis* (*my*, orange). **d** Schematic illustration of the octopod chromosome evolution history. The top segments are assumed to be the chromosomes of the ancestral cephalopods that phylogenetically closest to the nautilus, while the bottoms are the chromosomes of the last common octopod ancestors. The middle lines illustrate the chromosomal evolution process from ancestral cephalopods to the last common octopod ancestor. The line color corresponds to different chromosomes, and each line represents one synteny block between pairwise chromosomes

Coleoid cephalopods have a large number of karyotype (average *n* = 30 for octopus, *n* = 46 for squid) [24–26] and large genome size (average 3.69 Gb) than nautilus (*n* = 26; average genome size 0.76 Gb) (Additional file 1: Table s6). Understanding how the coleoid cephalopod genomes evolved from their ancestors would yield important insights into the cephalopod evolution. Here, we performed phylogenetic and chromosomal analyses to elucidate this question. We identified 585 single-copy orthologues from 28 genomes (including 25 molluscan species and 3 outgroups), constructed a maximum-likelihood phylogenetic tree, and further calibrated it using data available from the fossil record (Fig. 1a and Additional file 2: Fig. s2). The phylogenetic results reveal that coleoid cephalopods evolved from ancestral cephalopods at around 382 Ma, octopus and squid diverged at around 220 Ma, and *A. fangsiao* and *O. sinensis* diverged at approximately 44 Ma.

To elucidate how octopod genomes evolved, we performed chromosome-level synteny analyses among the octopod, nautilus, and scallop genomes. Briefly, we identified the homologous genes among pairwise species of *N. pompilius*, *A. fangsiao*, or *O. sinensis* (Additional file 2: Fig. s3–5), and the synteny blocks were generated using MCScanX if the chromosomal regions contain the same micro-syntenic blocks (> 3 consecutive genes) and gene orders (neglecting gene orientation) [28]. *A. fangsiao* and *O. sinensis* have 481 micro-synteny blocks distributed on 30 chromosomes of each species and show extensive collinearity with each other (occupying 83% and 86% of genome assembly, respectively) (Fig. 1c). As the two species show relative distant phylogenetic distances (diverged at 44 Ma, Fig. 1a), the high conservation of their chromosome reveals a conserved chromosome organization during the evolution process from the last common octopod ancestor to their descendants. Besides, we detected a less but conserved collinearity between octopod and squid genome, occupying 13% of *A. fangisao* genome assembly (on 30 chromosomes) and 7% of *Euprymna scolopes* genome assembly (on 190 contigs) (Fig. 1c). However, fewer synteny blocks are detected between octopod and nautilus, which is found only in 24 (out of 30) octopod chromosomes and 23 (out of 26) nautilus chromosomes (Fig. 1b, c), occupying 10.1% of octopus genome. As nautilus regarded as the closest extant lineage to coleoid cephalopods (see the “Background” section), the less conservation of chromosome between nautilus and octopods leads support to the extensive genome organization during the evolution process from the ancestral cephalopods to the last common octopod ancestor.

To further investigate how the karyotype of the last common octopod ancestors evolved, we reconstructed the evolutionary history of octopod genome (Fig. 1b, c). We assumed that the nautilus genome was less derived relative to the initial state of cephalopods (see the “Background” section), and the chromosomes of which were hypothesized to be retained in octopod lineages if the nautilus genome shared synteny blocks with both *A. fansiao* and *O. sinensis*. The results demonstrated that the increase of chromosome number in octopod clade is not only due to fission/fusion events, but also involved in chromosome loss/gain. We detected a total of 31 fissions of 17 nautilus chromosomes, and 30 subsequent fusions of 15 chromosomes (Fig. 1d). During the octopod chromosome evolution, we also detected 2 chromosome losses (Chr 2 and 22) in nautilus, and 6 chromosome gains (Chr 23, 25, 26, 28, 29 and 30) in the last common octopod ancestors (Fig. 1d). For squids, another taxon in coleoid cephalopods, they had no synteny blocks with the two lost chromosomes in nautilus based on the chromosome synteny analyses, but contained genome segments related to the 6 gained chromosomes in octopods (Fig. 1c), proving the chromosome gain or loss is essential events during the chromosomal evolution of coleoid cephalopod. As for the origin of the 6 gained chromosomes in octopods, they do not have any synteny blocks with the other chromosomes in both *A. fansiao* and *O. sinensis* (Additional file 2: Fig. s6), excluding the possibility that the 6 gained chromosomes were derived from genome duplication.

The genome size of coleoid cephalopods is nearly 5 times larger than that of *N. pompilius* (Additional file 1: Table s6). The expansion of genome size in celoid cephalopods is remarkable compared with the difference of karyotypes between the coleoid and nautilus (5 times *vs* 1.46 times). This is mainly due to the burst of genome repeats [2, 7, 10, 13], as the average repeat content in coleoid cephalopods is 48.72 ± 10.20% (1.58 ± 0.63 Gb, *N* = 11) (Additional file 1: Table s6). To reduce the impact of annotating methods on the results, we re-annotated the repeat contents of *O. sinensis*, *O. bimaculoides*, and *Architeuthis dux* using the same method (Additional file 1: Table s7). DNA-transposons are the most abundant repeat types (average 25.78%), followed by long interspersed nuclear elements (LINE, average 18.66%), and long-terminal repeats (LTR, average 11.06%) (*N* = 5; Additional file 1: Table s8). The contents of the repeat elements on the 6 gained chromosomes (see above) of *A. fangsiao* are 66.0 ± 1.5% (repeat length/chromosome length), which is similar to those on other chromosomes (63.2 ± 1.1%).

### Evolution characteristics of the expanded gene families

**Fig. 2:**
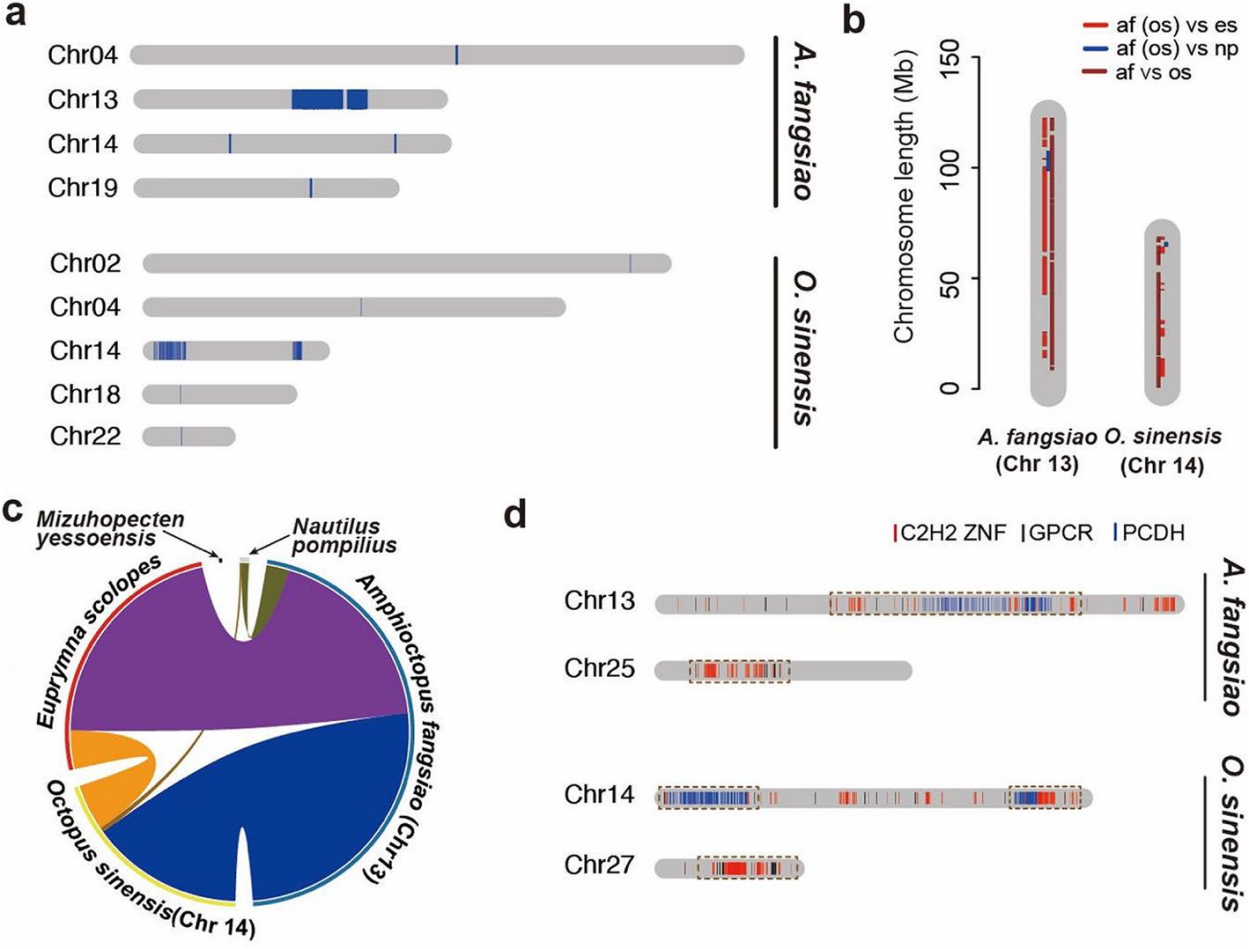
Genomic organization of the octopod expanded genes. **a** Chromosomal organizations of the protocadherin genes in *A. fangsiao* (top) and *O. sinensis* (bottom). **b** Synteny analyses between the octopod PCDH-clustered chromosomes (that is chromosome 13 in *A. fangsiao* and chromosome 14 in *O. sinensis*) and chromosomes of other species. Synteny blocks between pairwise species are labeled in colors: red for octopod (*A. fangsiao*, *af*; *O. sinensis*, *os*) and *E. scolopes* (*es*) comparison; blue for octopod (*A. fangsiao*, *af*; *O. sinensis*, *os*) and *N. pompilius* comparison; brown for *A. fangsiao* and *O. sinensis* comparison. The picture is plotted in R platform v4.1.2. **c** Comparison of the synteny blocks between the octopod PCDH-clustered chromosomes (that is Chr 13 for *A. fangsiao*, and Chr 14 for *O. sinensis*) and chromosomes of other species. The inner colored blocks represent synteny blocks between pairwise species, and the arch length represents the length of synteny block in individual species. The outer segments are chromosomes of each species: blue for *A. fangsiao*; yellow for *O. sinensis*; red for *E*. *scolopes*; black for *M. yessoensis*; gray for *N. pompilius*. The outer segments are only used for comparison of synteny block length and number, without chromosomal meaning. The picture is plotted using Circos v0.69 [29]. **d** The collinear modes between the gene families in octopus: PCDH and c2h2 zinc finger (C2H2 ZNF) on chromosome 13 of *A. fangsiao* and chromosome 14 of *O. sinensis*; C2H2 ZNF and G protein-coupled receptors (GPCR) on chromosome 25 of *A. fangsiao* and chromosome 27 of *O. sinensis*

**Fig. 3:**
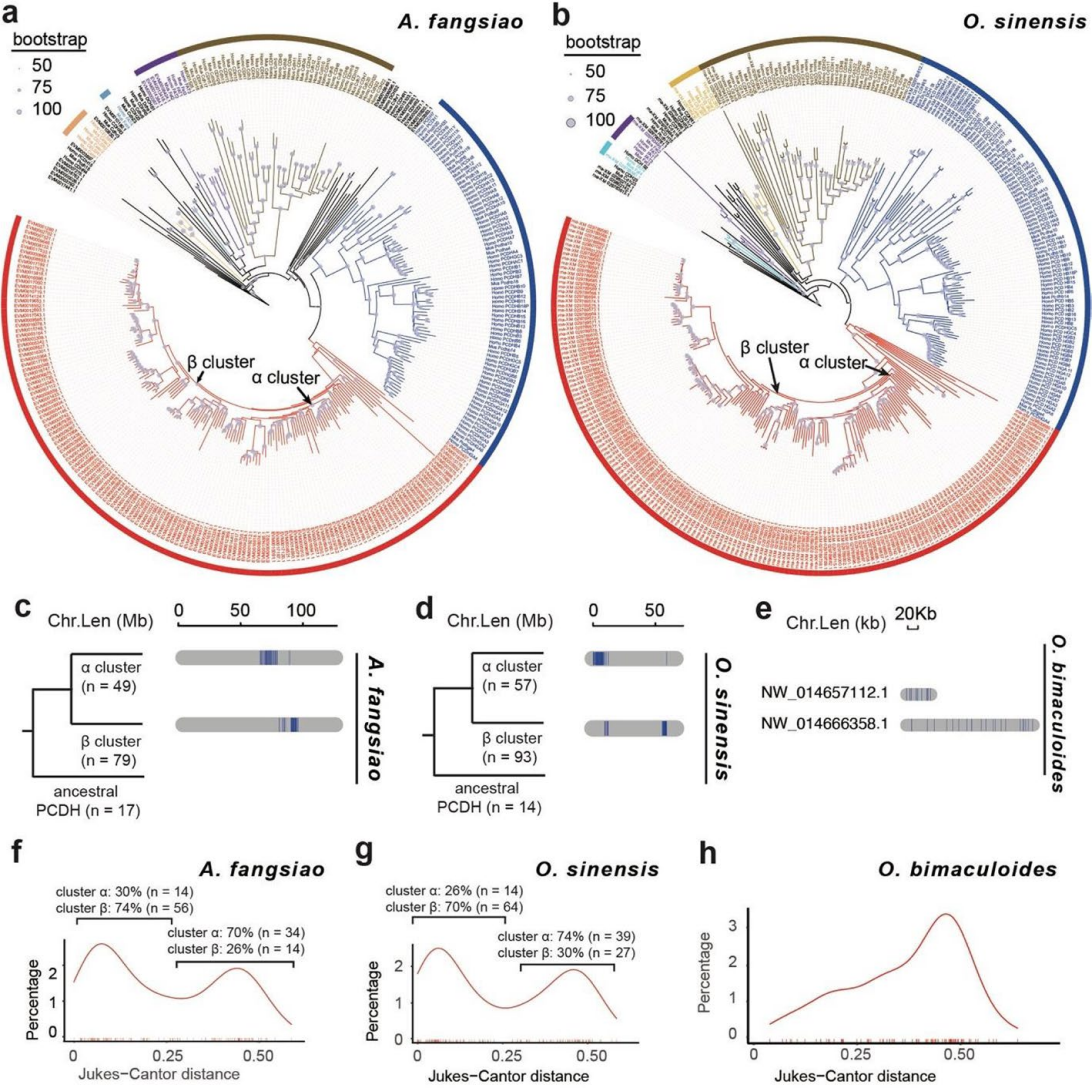
Phylogenetic analyses of the protocadherin (PCDH) genes in octopus. Maximum-likelihood phylogenetic tree of cadherin genes in *A. fangsia*o (**a**) and *O. sinensis* (**b**). The octopod protocadherin (PCDH) genes occupy an octopod-specific clade in the phylogenetic tree of both species, which are divided into three groups: the ancestral genes and two subsequent divisions (termed cluster α and β). **c** The two groups of PCDH in the phylogenetic tree correspond to the two separate clusters in chromosome 13 of *A. fangsiao* (**c**) and chromosome 14 of *O. sinensis* (**d**). **e** Two PCDH clusters in *O. bimaculoides*. The density plot of Jukes–Cantor distances for PCDH genes in *A. fangsiao* (**f**), *O. sinensis* (**g**), and *O. bimaculoides* (**h**). The ratios of α- or β-clustering PCDH genes fallen into each peak are listed over each peak

We identified the candidates of protocadherin, GPCR, and C2H2 ZNF genes using a hidden Markov model (HMM)-based method and also applied phylogenetic clustering method to separate protocadherin genes from other cadherin genes. From an overall view, C2H2 ZNF and GPCR genes are scattered on multiple chromosomes while protocadherin genes are clustered on a single one (Additional file 2: Fig. s7a). We identified 149 and 161 protocadherin genes in *A. fangsiao* and *O. sinensis*, which is consistent with the findings in *O. bimaculoides* (*N* = 168) [13]. The protocadherin genes can be divided into three separate phylogenetic groups (see below, Fig. 3a, b) and are distributed in cluster on a single chromosome (chromosome 13 in *A. fangsiao*, and chromosome 14 in *O. sinensis*) (Fig. 2a). The protocadherin-clustering chromosomes in *A. fangsiao* and *O. sinensis* show high collinearity with each other and with squid genome, yet both have only one small synteny block with *N. pompilius* (Fig. 2b, c). This indicates the octopod protocadherin genes were expanded after coleoids evolved from ancestral cephalopods. Apart from protocadherin, we also find clusters of C2H2 ZNF genes in both species, with four clusters of C2H2 ZNF genes on chromosomes 13, 25, and 29 in *A. fangsiao*, and four clusters on chromosomes 14, 27, 28, and 30 in *O. sinensis* (Additional file 2: Fig. s7b, c). Besides, we detected several collinear modes in the chromosomal distribution between C2H2 ZNF and protocadherin (or GPCR) (Fig. 2d). For example, some protocadherin and C2H2 ZNF genes are distributed closely on chromosome 13 (Chr13) in *A. fangsiao*, and on chromosome 14 (Chr14) in *O. sinensis*. C2H2 ZNF genes also have close chromosomal distances with GPCR genes on chromosome 25 (Chr25) in *A. fangsiao*, and on chromosome 27 (Chr27) in *O. sinensis*. The expanded genes in *A. fangsiao* and *O. sinensis* show high consistency in both contents and chromosome distributions, and this indicates that these gene families have already been expanded before the diverging of octopod species.

The protocadherin genes could be divided into three clusters using the phylogenetic clustering method: the ancestral genes and two subsequent divisions (termed cluster α and β PCDH) (Fig. 3a, b). Strikingly, the α and β PCDH groups in the phylogenetic tree correspond to two individual clusters on chromosome 13 of *A. fangsiao* (Fig. 3c) and chromosome 14 of *O. sinensis* (Additional file 2: Fig. 3d), which is supported by the clustering distribution of protocadherin genes in *O. bimaculoides* (Fig. 3e) [13]. To further investigate when this gene family expansion happened, we calculated the divergence time of protocadherin and C2H2 ZNF genes using a Jukes-Cantor distance-based method. Divergent time analyses reveal that there was a common expansion of octopod protocadherin genes at around 65 Ma, coinciding with the Cretaceous-tertiary Extinction, and a recent burst was detected in some octopods (i.e., *A. fangsiao* and *O. sinensis*) at around 8–14 Ma (Fig. 3f-h). However, the genes in the α and β PCDH groups do not expand in a parallel scenario: most of α PCDHs were expanded at the first expansion (around 65 Ma; covering 70% of total α PCDH in *A. fangsiao* and 74% in *O. sinensis*), while β PCDH expansion mostly happened in a recent time (8–14 Ma; covering 74% of total β PCDH in *A. fangsiao* and 70% in *O. sinensis*). Besides, the C2H2 ZNF expansion occurred at around 41 Ma, which is between the time of two PCDH expansion (Additional file 2: Fig. s8). Collectively, these results support a possible evolution scenario that there might be a first PCDH expansion in the last common octopod ancestor, and a second expansion after the octopod division.

### Functional patterns of the expanded gene families at cellular level

**Fig. 4:**
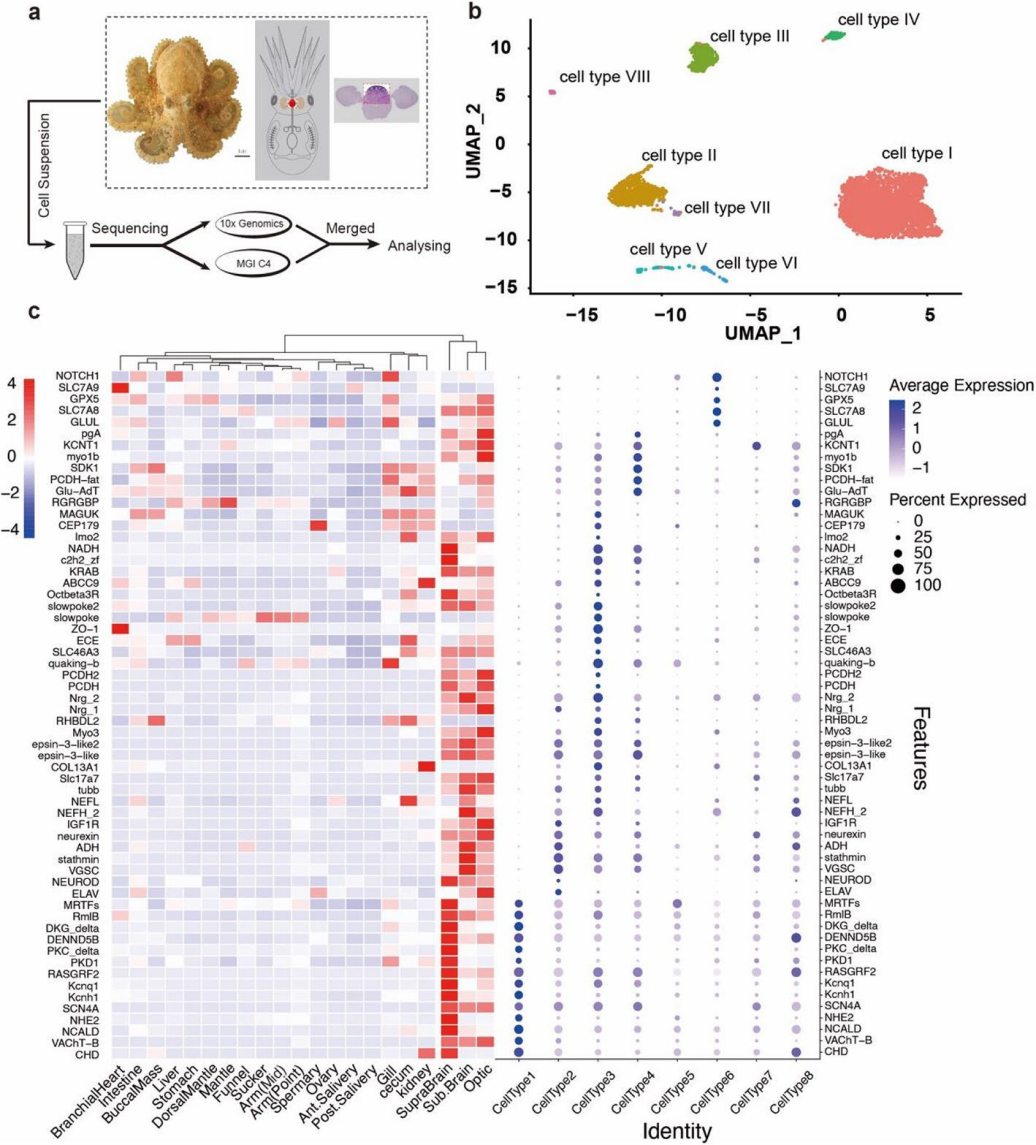
Single-nucleus RNA sequencing (snRNA-seq) profiles of the supra-esophageal brain (sup-brain) of *A. fangsiao*. **a** Experimental protocol of snRNA-seq of *A. fangsiao* supra-esophageal brain. The supra-esophageal brain is labeled in red dashed circle. **b** Uniform Manifold Approximation and Projection (UMAP) representation of snRNA-seq profiles of the supra-esophageal brain of *A. fangsiao* (*N* = 5,011 cells). Cells were merged from 10x Genomics and DNBelab C4 data. **c**, Expression of the top ten marker genes of cell types in bulk transcriptomic (left) and snRNA-Sseq (right) data

We performed single-nucleus RNA sequencing (snRNA-seq) of the supra-esophageal brain of *A. fangsiao* to investigate the cellular heterogeneity in the octopod supra-esophageal brain and the expression patterns of protocadherin, GPCR, and C2H2 ZNF at cellular level. To ensure the accuracy of the results, we applied two snRNA-seq methods: 10x Genomics and DNBelab C4 (Fig. 4a). We obtained transcriptomic profiles of 3754 cells using 10x Genomics method and another 1402 cells using DNBelab C4 method. The results of cell clusters derived from the two methods are consistent, and both contained 8 cell types (Additional file 2: Fig. s9; Additional file 1: Table s9), indicating the accuracy of the sampling and sequencing methods.

We identified a total of 8 cell types in the supra-esophageal brain of *A. fangsiao* (Fig. 4b), and 2434 cluster marker genes based on the cellular transcriptome dynamics (Fig. 4c and Additional file 2: Fig. s10; Additional file 1: Table s10). These cluster marker genes reflect the functional differences in each of the supra-esophageal brain cells (or regions); we then performed a functional enrichment analysis to investigate the cellular heterogeneity of octopod supra-esophageal brain. The KEGG enrichment results indicated that cell types II, III, IV, and VII have similar functions in signal transduction (i.e., Rap1, adrenergic, cAMP, and cGMP-PKH signaling pathway), which is different from cell types V and VI that both are enriched in similar functions of cell binding modules (i.e., cell adhesion, focal adhesion, regulation of action cytoskeleton and tight junction) (Additional file 2: Fig. s11). We also analyzed whether there are expression biases of the cell marker genes of the supra-esophageal brain in the bulk transcriptomic data (Additional file 2: Fig. s12). Usually, the tissue-specific genes can reflect the functional differences among tissues [30], and we analyzed the functional relationships between cell types of the supra-esophageal brain and other tissues, which is mainly based on the expression analyses of cell marker genes in the bulk transcriptomic data. The results show that the cell marker genes of seven (out of eight) cell types in supra-esophageal brain are highly expressed in bulk transcriptomic data of sub-esophageal brain and optic lobes, indicating functional relationships between supra-esophageal brain and sub-esophageal brain (or optic lobes). Notably, there are six cell types of supra-esophageal brain whose marker genes are highly expressed in the bulk transcriptomic data of gills, especially cell type V with eight (out of the top ten) marker genes. The function relationships between supra-esophageal brain and gill tissue suggest a potential group of cells in supra-esophageal brain controlling the gill functions (i.e., respiration, circulation, and excretion).

To further identify the cell types in the supra-esophageal brain, we used marker genes collected from both model organisms and octopods (Additional file 1: Table s11) and identified three cell types (Fig. 4c). Vesicular acetylcholine transporter (*VAchT*) mediates transfer of acetylcholine (*Ach*) from the cytoplasm into synaptic vesicles and is employed as a marker for cholinergic neurons [31]. A choline dehydrogenase gene (EVM0000404.1, *CHD*) and a vesicular acetylcholine transporter-B gene (EVM0001846.1, *VAChT-B*) were highly expressed in cell type I; we thus estimated cell type I as cholinergic-like neurons (Fig. 4c). In cell type II, the neuron marker *NEUROD* and embryonic lethal abnormal visual system (*Elav*) was highly expressed; we thus designated cell type II as *Elav*-like neurons. In cell type III, we observed the high expression of three neurofilament-related genes (two *NEFH* genes and one *NEFM* gene), one tubulin beta gene (*tubb3*) gene, and one vesicular glutamate transporter 1-like gene (*VGluT*). This indicated that cell type III might be a glutamatergic-like neuron. Several cell-adhesion modules (i.e., protocadherins; and neuroglian-like, *nrgs*) were highly expressed in these glutamatergic-like neurons, which may facilitate cell-to-cell interactions at synaptic contacts.

Given the commonly high expression of the protocadherin, GPCR, and C2H2 ZNF genes in the neural system, we ask whether there are any functional relationships (i.e., positive enhancement, or negative complementation) among these genes. Among the cluster marker genes (*N* = 2434) of the supra-esophageal brain, we detect 72 protocadherin (48.32% of all protocadherin), 61 GPCR (21.11% of all GPCR), and 27 C2H2 ZNF genes (2.90% of all C2H2 ZNF) (Additional file 1: Table s12). The GPCR marker genes (*N* = 61) are mainly in cell type II (*N* = 24 *vs* average 5 in others), and the protocadherin marker genes (*N* = 72) are in cell types II (*N* = 21), III (*N* = 16), and VII (*N* = 16) (Additional file 2: Fig. s13, 15). We calculated the average expression of protocadherin, GPCR, and C2H2 ZNF genes in cells using a function *AverageExpression* of Seurat v4.0.6, and compared the gene expression in cell types I–VIII (Additional file 1: Table s13-18). Results indicated that the per-cell expression of the expanded genes was different in cell types. The expression of protocadherin genes was similar in cell types I, II, III, and VII (*P* > 0.05, Wilcoxon signed-rank test) but higher than that in other cell types (*P* < 0.05, Wilcoxon signed-rank test); meanwhile, GPCR genes are also highly expressed in cell type II (*P* < 0.01, Wilcoxon signed-rank test) (Additional file 2: Fig. s14 and 15). As described above, the cell types I, II, and III are three putative neural type cells; the co-expression of protocadherin and GPCR in neural cells might facilitate the neural development and signal transduction of the brain, which is consistent with the findings in other cephalopod species [32–34].

## Discussion

The karyotypes of most squids, octopuses, and nautiluses are 26, 30, and 46 chromosomes, respectively [24, 27], indicating an increase of chromosome number in coleoid cephalopods since their origin from the ancestral cephalopods. A primitive hypothesis of whole-genome duplication in coleoid cephalopods was proposed based on the chromosome numbers [35, 36], but has been rejected by *Hox* gene [11, 13, 37], micro-synteny [13], and macro-synteny [10] analyses. Due to the limited genome data of recently diverged or intermediate species, it is difficult to elucidate how the karyotype of coleoid cephalopod evolved from their ancestors. However, as the conserved synteny blocks among species can reflect the lineage-specific evolutionary history [38], we can trace some clues through the comparative synteny analyses on the genomes of *Nautilus pompilius*, *O. sinensis*, and *A. fangsiao*, three available cephalopod genomes with chromosomal scale. The synteny analyses revealed a less collinear signature between octopods and nautilus chromosomes, suggesting extensive genome rearrangements occurring during the evolution of ancestral octopods. This corresponds to the observation that an intense, early genome reorganizations occurred before the split of major coleoids [39]. Macrosynteny-based karyotype analyses further elucidate a putative evolutionary scenario describing how ancestral octopod chromosomes evolved from an ancestral state. However, some results still need deep analyses combined with more cephalopods that are keynotes in phylogeny, such as how the chromosome gain events happened in the evolutionary process.

Coleoid cephalopods show lineage-specific expansions of protocadherin, GPCR, and C2H2 ZNF [13], yet the gene families of which are not expanded in nautilus [2, 12]. As for the origin and role of these expanded gene families, several micro-synteny analyses have been performed [13, 40, 41], yet the chromosome-level gene family analyses are still lacking. Here, we conducted comparative genomic analyses with three chromosome-level genomes: *N. pompilius* [12], *O. sinensis* [6], and *A. fangsiao*, to explore how protocadherin, GPCR, and C2H2 ZNF genes in coleoids. The results revealed tandem duplications of these expanded gene families on chromosomes and also suggested collinear modes between pairwise genes. These distribution characteristics are similar to the results in *O. bimaculoides* [13] and *E. scolopes* [10] which exhibit a more comprehensive perspective at the chromosome level. Studies have shown that the cephalopod genomes have experienced extensive restructurings, leading to many tightly linked, evolutionary unique gene clusters [42], confirming the observation of collinear modes between coleoid expanded genes in the present study. Besides, as the genomic location of genes can influence their expressions [43], the adjacent genomic locations between pairwise expanded genes suggest a possible co-regulation scenario by using similar transcription elements.

Tandem-duplicated protocadherin genes are observed on one chromosome in two octopods, *A. fangsiao* and *O. sinensis*, which is consistent with a previous study that has revealed the tandem duplication of protocadherin genes on two scaffolds (*n* = 31 and 17 of total 169) in *O. bimaculoides* genome [13]. Phylogenetic analyses reveal two separate expansions of protocadherin genes: one is estimated to happen in the last common octopod ancestor, and another is after the octopod divergence. Except for a few representatives (i.e., *Hox* genes), the role of clustered genes in species development and evolution still needs further elucidation. Here, we find the commonly high expression and co-expression of the protocadherin and GPCR genes in specific neuron cells. As the protocadherin genes can mediate homophilic intercellular binding by forming multimers within a cell [44], the combination of GPCR and protocadherin in neural cells may contribute to the signal transductions between cells.

## Conclusions

In conclusion, we provide a chromosome-level genome and a single-nucleus profile of the supra-esophageal brain for *A. fangsiao*. One important contribution of this study is that we performed the chromosome-level synteny analyses between nautilus and octopod genomes, which led to the discovery of the chromosome rearrangement patterns (i.e., chromosome fission, fusion, gain, and loss) during the octopod chromosome evolution. These findings add evidences on how coleoid cephalopod genomes evolved from ancestral cephalopods, which was not only due to the chromosome fission/fusion, but also related to the chromosome loss/gain.

## Methods

### Genome sequencing, assembly, and annotation

The wild and mature individuals of *A. fangsiao* were collected in Lianyungang (N 34°, E 119°, Jiangsu province, China), and species identity was validated by the sequencing of the mitochondrial COI gene (UJY97108). The octopuses were temporarily maintained in a 2-L sea-water tank at 18°C as described before [45], and individuals were anesthetized using MgCl_2_ (>10 g/L) before use. The muscle of arms was used for genome sequencing. DNA extraction was performed by using a modified version of the cetyl trimethyl-ammonium bromide (CTAB) method [46]. The concentration and purity were detected using a NanoDrop spectrophotometer, and the integrity of DNA was assessed by pulsed-field electrophoresis. The large segments of DNA were filtered using the BluePippin System and then used to construct ONT library. The high-quality library was sequenced on the ONT PromethION platform. The clean data was de novo assembled using Canu v1.5 [47] after filtering. The draft genome was assembled using wtdbg2 [48]. To improve the quality of genome assembly, we performed three rounds of error correction using ONT long-read data by Racon v1.3.1 [49], and three rounds of polishing using Illumina short-read data by Pilon v1.22 [50].

To get a chromosomal-level genome assembly, we performed Hi-C sequencing [51]. Fresh mantle muscle tissue was fixed using formaldehyde with a final concentration of 1%. After reversal of the cross-links, ligated DNA was purified and sheared to a length of 300–700 bp. Biotinylated DNA fragments were captured with streptavidin beads and used for Hi-C fragment library construction. High-quality Hi-C libraries were sequenced on Illumina HiSeq X platform. To obtain uniquely mapped read pairs, the raw data were aligned to the initial genome assembly using BWA-MEM v0.7.10 [52]. Hi-C pro software [53] was used to evaluate the Hi-C data. The valid read pairs were used for draft genome correction and chromosome-level genome assembly. We aligned the raw reads to the genome assembly using bowtie2 v.2.2.5 [54] and built raw inter/intra-chromosomal contact maps after filtering out the low-quality reads. We anchored the contig sequences into 30 chromosomes using Juicer v.1.5 [55] and 3D-DNA pipeline v.170123 [56].

The tandem repeat sequences were predicted using TRF v4.09 [57]. The long terminal repeats (LTR) were predicted using LTR_FINDER.x86_64-1.0.6 [58]. Transposable elements (TEs) were predicted using two methods: homolog-based and de novo-based prediction. Novel repeats were predicted using RepeatModeler (http://www.repeatmasker.org). RepeatMasker v3.3.0 was used to identify the known TEs. The consensus and non-redundant library were obtained by the combination of known, novel, and tandem repeats. We re-annotated repeat sequences of *O. sinensis*, *O. bimaculoides*, *A. dux*, and *E. scolopes* using the same method as described above.

The protein-coding genes were annotated using three methods: de novo, homolog-based, and transcriptome-based. We performed de novo gene annotation using Augustus v2.4 [59], GlimmerHMM v3.0.4 [60], SNAP [61], Geneid v1.4 [62], and GeneScan [63]. The homolog-based annotations were performed using GeMoMa v1.3.1 [64] based on the homologous peptides from *Danio rerio*, *O. bimaculoides*, *O. sinensis*, and *Larimichthys crocea*. Twenty-one adult tissues/organs of *A. fangsiao* were chosen for transcriptome sequencing. These RNA-seq data were aligned to the genome using HISAT v2.0.4 (--max-intronlen 20000, --min-intronlen 20), transcripts were assembled using StringTie v1.2.3 [65], and the gene structures were predicted using TransDecoder v2.0 (http://transdecoder.github.io). PASA v2.0.2 [66] was used to identify and analyze unigenes. Finally, genes predicted from the above methods were merged to a consensus gene set using EVM v1.1.1 [67] and modified by PASA v2.0.2 (-align_tools gmap-maxIntronLen 20000) [66].

The functional annotation of the predicted genes was performed by homology searching in several public gene databases, including NCBI-NR, TrEMBL [68], KOG [69], GO [70], and KEGG [71] using BLASTp (identities ≥ 50% and *E*-value ≤ 1e−05). We used tRNAscan-SE [72] to identify the tRNAs in the genome. MicroRNA and rRNA were identified by searching homology against the miRBase (http://www.mirbase.org) and Rfam database (http://rfam.xfam.org/) using Infenal v1.1 (http://infernal.janelia.org/). Pseudogenes were annotated based on the homology-searching using GenBlastA v1.0.4 [73] and verified using GeneWise v 2.4.1 [74].

### Sample collection and single-nucleus suspend preparation for the supra-esophageal brain of *A. fangsiao*

Alive, mature animals of *A. fangisao* were anesthetized using 7% MgCl_2_, and the supra-esophageal brain was physically separated and immediately digested in a mixture of 0.025% trypsin, DMEM, and 30‰ artificial sea salt (pH = 8.2) at 20°C for 10 min. The cells were screened using 40-mm cell strainers, washed using 30‰ artificial sea salt (pH = 8.2) and 0.5% BSA, centrifugated under a condition of 500g and 10 minutes, and finally resuspended in a mixture of 30‰ artificial sea salt (pH = 8.2) and 0.5% BSA. The prepared cells were used for constructing single-nucleus RNA sequencing (snRNA-seq) library with two methods: Chromium single cell 3 prime v2 reagent kit (10x Genomics) and DNBelab C4 scRNA-seq kit (MGI). The libraries derived from 10x Genomics were constructed according to the manufacturer’s instructions. The DNA nanoballs (DNBs) were sequenced on the BGISEQ-500 sequencing platform with a paired-end read length of 28+100 bp. For the MGI method, barcoded mRNA capture beads, droplet generation oil, and the single-cell suspension were loaded into the corresponding reservoirs on chip for droplet generation for 20 min. The droplets were gently removed to the collection vial and placed at room temperature for 20 min. Droplets were then broken and collected by a bead filter (MGI). The supernatant was removed, and the bead pellet was resuspended in 100 μl RT mix. The mixture was then thermal cycled as follows: 42 °C for 90 min, 10 cycles of 50 °C for 2 min, 42 °C for 2 min. Afterward, the PCR master mix was added to the beads pellet and thermal cycled as follows: 95 °C for 3 min, 17 cycles of 98 °C for 20 s, 58 °C for 20 s, 72 °C for 3 min, and finally 72 °C for 5 min. Amplified cDNA was purified using 60 μl of AMPure XP beads. The cDNA was subsequently fragmented to 400–600bp with NEBNext dsDNA Fragmentase (New England Biolabs) according to the manufacturer’s protocol. Indexed sequencing libraries were constructed using the reagents in the DNBelab C4 scRNA-seq kit following the steps: (1) post fragmentation size 1 selection with AMPure XP beads, (2) end repair and A-tailing, (3) adapter ligation, (4) post ligation purification with AMPure XP beads. The sequencing libraries were quantified by Qubit (Invitrogen). The DNA nanoballs (DNBs) were loaded into the patterned nanoarrays and sequenced on the MGISEQ-2000 sequencer using the following read length: 41 bp +100 bp.

### Data processing of single-nucleus transcriptomic data

The raw FASTQ files were processed to generate a gene-barcode matrix using CellRanger v2.0.1 pipeline. The downstream analyses were based on *Seurat* pipeline. Briefly, we first discarded cells that expressed less than 200 genes, and genes expressed in less than three cells. Only cells with 200–2500 expressing genes and <5% of mitochondrial genes were retained for further analyses. The UMI (unique molecular identifier) counts of each cell were normalized using the function *NormalizeData* with the parameters *normalization.method* set to *LogNormalize*, and *scale.factor* set to 10,000. To select the variable genes, we applied the function *FindVariableFeatures* with the parameters *selection.method* set to *vst*, and *nfeatures* set to *2000*. To remove possible data bias, we regressed the UMI counts data using the function *ScaleData* with the parameter *features* set to *all.genes*. The selected genes were then used to perform a principal component analysis (PCA) using the function *RunPCA*, and the top 20 PCs were tested for significance using the function *JackStraw* and *ScoreJackStraw*. To calculate the neighborhood distance of pairwise cells, we built the SNN on the first ten principal components using the function *FindNeighbors*. The marker genes of cell clusters were identified using the function *FindClusters* with a resolution of 0.6. Dimension reduction was conducted with a Uniform Manifold Approximation and Projection (UMAP) method using the function *RunUMAP*. To identify differentially expressed genes (DEG) in each cell type, we used the function *FindAllMarkers*. The selected DEGs were used for plotting, such as comparing gene expressions across cell types using the function *DotPlot*, comparing functional enrichments in different cell types using the function *compareCluster* within *clusterProfiler* v4.3.1 package [75]. To investigate the functional relationships of supra-esophageal brain and other tissues, the bulk transcriptomic data of DEGs were also used to create a heatmap plot using *pheatmap* package.

### Phylogenetic analyses

We performed comparative genomic analysis with a total of 28 genomes, including *Bathyacmaea lacteal* [76], *Lottia gigantea* [77], *Chrysomallon squamiferum* [78], *Lanistes nyassanus* [79], *Marisa cornuarietis* [79], *Pomacea canaliculate* [79], *Biomphalaria glabrata* [80], *Aplysia californica* GCF_000002075.1, *Elysia chlorotica* [81], *Argopecten purpuratus* [82], *Pecten maximus* [83], *Mizuhopecten yessoensis* [38], *Anadara broughtonii* [84], *Crassostrea gigas* [85], *Saccostrea glomerata* [86], *Mytilus coruscus* [87], *Lutraria rhynchaena* [88], *O. bimaculoides* [13], *O. sinensis* [6], *H. maculosa* [9], *E. scolopes* [10], *A. dux* [11], *N. pompilius* [2], *A. fangsiao*, *Acanthopleura granulate* [89], *Phoronis australis* [90], *Capitella teleta*, and *Helobdella robusta* [77]. We identify single-copy orthologous genes using Orthofinder 2.5.2 [91] with default parameters and retained the orthologs sampled in at least 18 taxa (≥ 2/3 of total taxa). A total of 585 orthologous genes were aligned using MUSCLE v3.8.31 with default parameters [92], and trimmed using trimAl v1.4 with the option “*-automated1*” [93]. All alignments were combined into one supergene using PhyloSuite [94] on a windows platform. The phylogenetic analysis was conducted using IQtree v2.1.2 [95] with 1000 replicates and the parameter of -MFP to automatically select the best fit model for each partition, and using PhyloBayes (pb_mpi 1.8c) [96] with the parameter “-*cat -gtr -dgam 4 -dc*”. The divergence time was estimated using MCMCtree in PAML v4.9 [97]. The fossil records used were as follows: a hard minimum bound of 168.6 Ma and a soft maximum bound of 473.4 Ma for the divergence of the *Aplysia* and the *Biomphalaria* [98]; a hard minimum bound of 470.2 Ma and a soft maximum bound of 531.5 Ma for the appearance of Gastropoda [98]; a hard minimum bound of 532 Ma and a soft maximum bound of 549 Ma for the first appearance of molluscs [99]; a hard minimum bound of 550.25 Ma and a hard maximum bound of 636.1 Ma for the appearance of Lophotrochozoa [99]. The best-fit model, LG+G4, was applied because this model was found to be the best model in 214 out of 585 partitions (36.58%), with the burn in and sampling frequency set to 10,000,000 and 1000, respectively.

### Chromosome analyses

To investigate the karyotype evolution history of last common octopod ancestor, we performed comparative synteny analyses among *A. fangsiao*, *O. sinensis* [6], and *N. pompilius* [12]. The longest protein of each gene was selected for homologous searching if there existed multi transcripts. We identified homologous sequences between pairwise species using DIAMOND [100] with the *p*-value cut-off set to 1E−5, and enabling the parameter “--sensitive”. Only the top hits were kept for further analyses. The identified gene pairs were used to construct chromosome collinearity matrix based on the general feature format (GFF) files. MCScanX [101] was further used to generate synteny blocks between pairwise chromosomes, and the blocks were plotted using Circos v0.69 [29]. To reconstruct the evolution history of octopod chromosomes, we gave definition of the chromosomal fission, fusion, loss, and gain with the assumption that nautilus represents a unique branch in cephalopods that can be regarded as the closest lineage to the common ancestor of coleoid cephalopods (see the “Background” section). If the chromosomes of the ancestral cephalopods have no synteny blocks with the last common octopod ancestors, these ancestral cephalopod chromosomes were assumed to be lost during the evolution process from the ancestral cephalopods to the last common octopod ancestors. If the chromosome C (assumed) of the ancestral cephalopods have synteny blocks with multiple chromosomes (2≤ n ≤30) of the last common octopod ancestor, chromosome C was assumed to experience n-1 fissions during the evolution process from the ancestral cephalopods to the last common octopod ancestors. Similarly, if multiple chromosomes (2≤ *n* ≤26) of ancestral cephalopod have synteny blocks with one chromosome of the last common octopod ancestor, the n chromosomes of the ancestral cephalopods were assumed to experience n-1 fusions during the evolution process from the ancestral cephalopod to the last common octopod ancestors.

### Gene family analyses

To identify protocadherin, G-protein coupled receptors (GPCR), and C2H2 superfamily of zinc-finger transcription factors (C2H2 ZNF), we used a hidden Markov model (HMM)-based method. We selected the longest protein of each gene for further analyses if there existed multi transcripts. The hidden Markov models (HMM) profiles of genes are downloaded from the Pfam website (http://pfam.xfam.org/). Based on the raw HMM profiles and proteomes, we performed homologous searching using the function *hmmsearch* of HMMER v3.3. The outputs were filtered with a *E*-value cut-off of 1E−20, and then aligned using MAFFT [102]. We constructed local HMM profiles using the function *hmmbuild* of HMMER v3.3.2 and re-performed homologous searching based on the local HMM profiles and proteomes. The sequences were further validated using PfamScan. To classify protocadherin among other cadherin genes, we applied a phylogenetic tree-based method. The cadherin genes of model species (i.e., human and mouse) were downloaded from the public database and aligned with octopod cadherin genes (*A. fangsiao* or *O. sinensis*) using MAFFT [102] with default parameters. The poorly aligned regions were removed using trimAl v1.4 [93] with the option “*-automated1*”. We constructed phylogenetic analyses of cadherin genes using IQtree v2.1.2 [95] with 1000 replicates and the parameter -MFP to automatically select the best fit model for each partition. The octopod genes adjacent to protocadherin genes of model species (i.e., human and mouse) were identified as octopod protocadherin genes. The results were modified in iTol [103].

To investigate the chromosomal distributions of protocadherin, GPCR, and C2H2 ZNF genes, we created a data matrix of gene coordinates based on the general feature format (GFF) files and plotted using the base packages in R v4.1.2. To date the burst of protocadherin and C2H2 ZNF genes, we applied a Jukes–Cantor correction method [13]. We identified the paralogous genes using DIAMOND v0.9.36.137 [100] with a *P*-value cut-off of 1E−5. The gene pairs were aligned using paraAT [104], and the adjusted Jukes–Cantor distances (JC) were calculated using *distmat*. The date was calculated using a formula: date = JC/2r, where JC is the adjusted Jukes–Cantor distances calculated above, and r is the substitution rates per site per million years [13].

## Supplementary Information

**Additional file 1: Table s1.** Statistics of Nanopore Clean Data. **Table s2.** Statistics of Nanopore Data after correction. **Table s3.** Comparisons of octopod genomes. **Table s4.** Gene information of *Amphioctopus fangsiao* genome. **Table s5.** Repeat statistics of *Amphioctopus fangsiao* genomes. **Table s6.** Comparison of genome size and repeat content in cephalopods. **Table s7.** Details of cephalopod repeats. **Table s8.** Repeat content in present study. **Table s9.** Percentage of cells that derived from two sequencing platforms in each cluster (before combining). **Table s10.** Cell markers in the octopod supra-brain. **Table s11.** Marker genes used in present study. **Table s12.** Counts of protocadherin, GPCR, and C2H2 ZNF genes in each cell cluster. **Table s13-18.** Average expression of protocadherin, GPCR, C2H2 ZNF genes in each cell cluster.

**Additional file 2: Fig. s1.** The genetic linkage map of Hi-C data. **Fig. s2.** Phylogenetic analyses based on ML and Bayesian inference methods. **Fig. s3-5.** Heat map of homologous genes in pairwise chromosomes of *A. fangsiao* and *O. sinensis* and *N. pompilius*. **Fig. s6.** Synteny analyses between *A. fangsiao*, *O. sinensis*, and *N. pompilius*. **Fig. s7.** Genomic organization of protocadherin, GPCR and C2H2 ZNF in *A. fangsiao* and *O. sinensis*. **Fig. s8.** Divergent time of C2H2 ZNF. **Fig. s9.** Cell population composition of the supra-esophageal brain. **Fig. s10.** Expression of top 10 marker genes of cell type 1-8. **Fig. s11.** Comparisons of functions in cell type 2-8. **Fig. s12.** Bulk transcriptomic expression of 10 marker genes. **Fig. s13.** Bar plot reflected the number of C2H2 ZNF (top), protocadherin (middle), and GPCR (bottom) genes in marker genes of cell type 1-8. **Fig. s14.** Heatmap of GPCR, C2H2 ZNF, and protocadherin (x axis) expressions in the sup-brain cell types (x axis). **Fig. s15.** Boxplot of average expression of protocadherin (a), GPCR (b), and C2H2 ZNF (c) in cells.

**Additional file 3: **Command line scripts used in present study. **Script 1**–phylogenetic and chromosomal analyses. **Script 2**–Chromosome evolution. **Script 3**–Gene family analyses. **Script 4**–Single cell analyses.

## Data Availability

The raw sequences used for *A. fangsiao* genome assembly and RNA-seq have been deposited in the NCBI Sequence Read Archive under the BioProject number be PRJNA762647 [105]: transcriptomic data (SRR22556679–SRR22556724), Hi-C data (SRR16115432), and genome assembly data (SRR16115480-SRR16115564, SRR16121316). The assembled genome, transcriptome, predicted transcripts, and proteins have been deposited in Figshare (https://figshare.com/s/fa09f5dadcd966f020f3) [106]. The raw data of single-nucleus RNA sequencing can be obtained from CNSA (CNGB Nucleotide Sequence Archive) by assembly ID: CNP0002082 [].
