# Supplementary material for: The gold-ringed octopus (Amphioctopus fangsiao) genome and cerebral single-nucleus transcriptomes provide insights into the evolution of karyotype and neural novelties
Source: BMC Biol. 2022 Dec 27;20:289. doi: 10.1186/s12915-022-01500-2 (PMC9795677; doi:10.1186/s12915-022-01500-2)

Fig. s1 | The genetic linkage map of Hi-C data. LG 01–30 are the abbreviations of Lachesis Group 01-30, and correspond to 30 pseudochromosomes. The blocks represent the contact between one location and the other locations. The color bar illuminates the contact density from orange (low) to red (high).

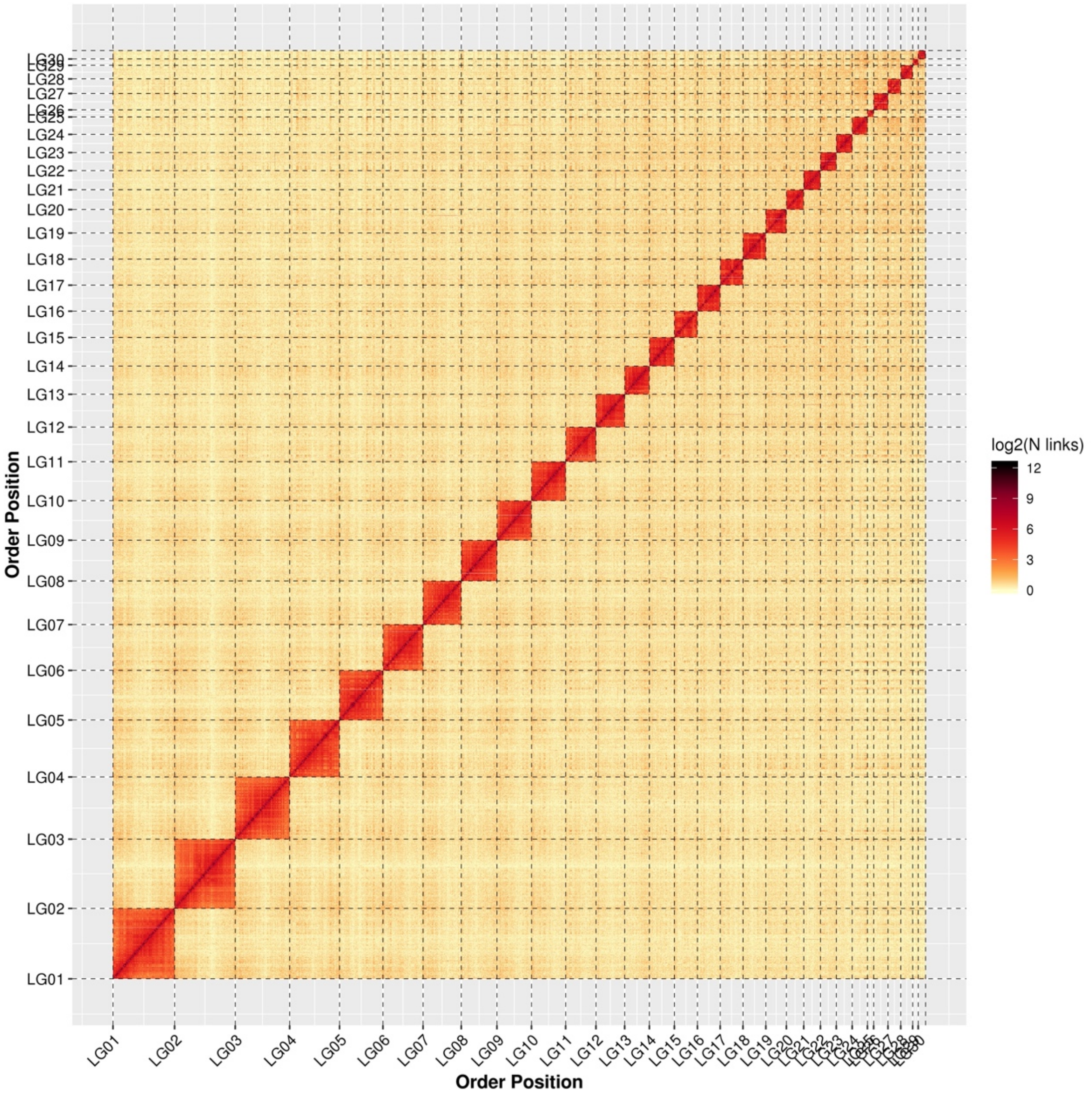

Fig. s2 | a) Maximum-likelihood tree of 28 genomes using IQtree v2.1.2 [89] an. ML-bootstrap/ultrafast-bootstrap values are listed at the right of each node. b) Bayesian phylogenetic tree of 28 genomes using PhyloBayes [90].

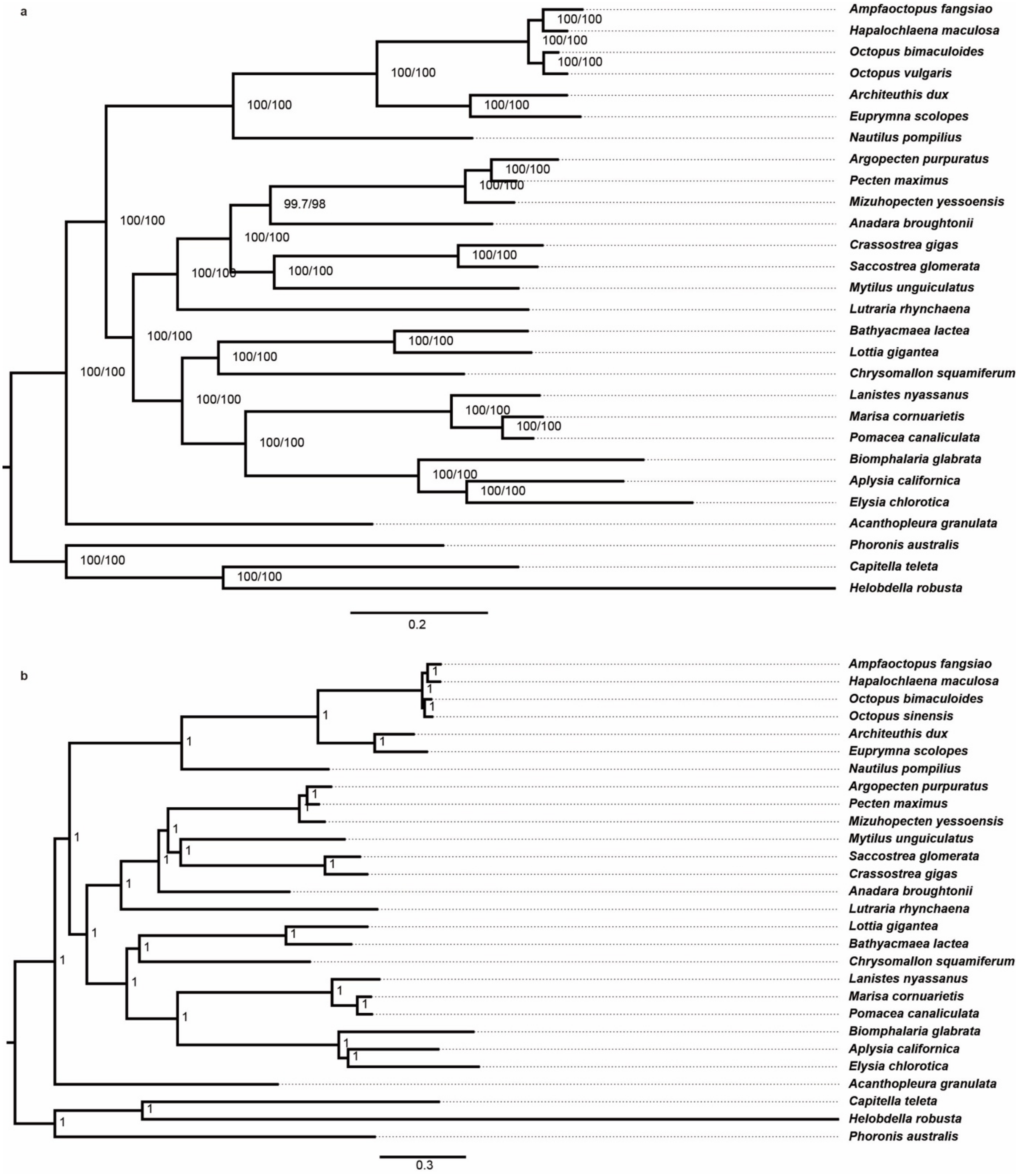

Fig. s3 | Heat map of homologous genes in pairwise chromosomes of *A. fangsiao* (ampfa) and *O. sinensis* (octsi). Each block represents the number of homologous genes.

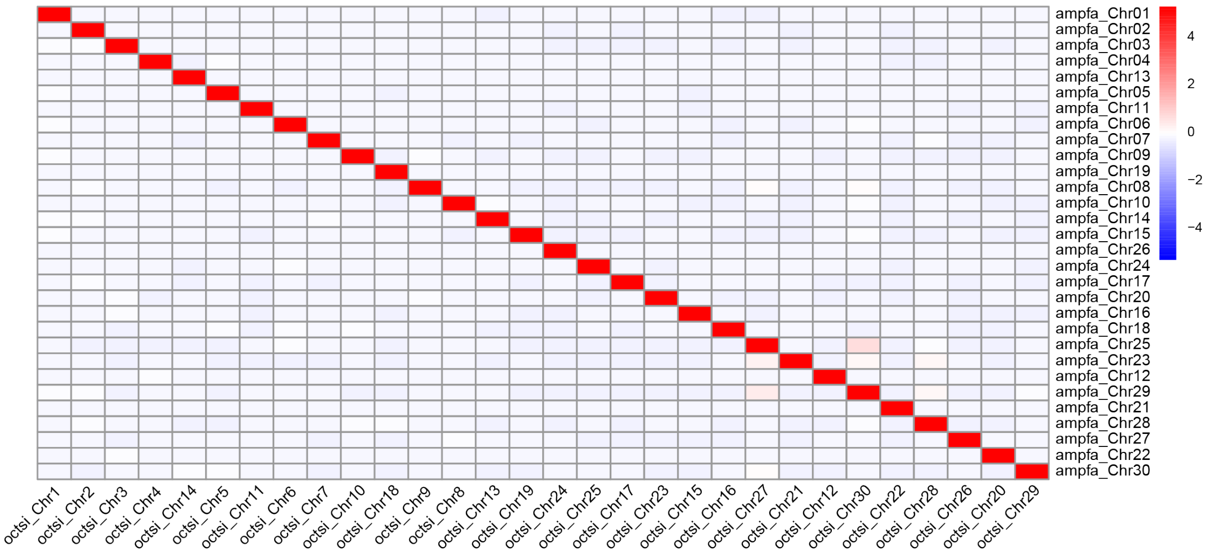

Fig. s4 | Heat map of homologous genes in pairwise chromosomes of *A. fangsiao* (ampfa_) and *N. pompilius* (naupo_). Each block represents the number of homologous genes.

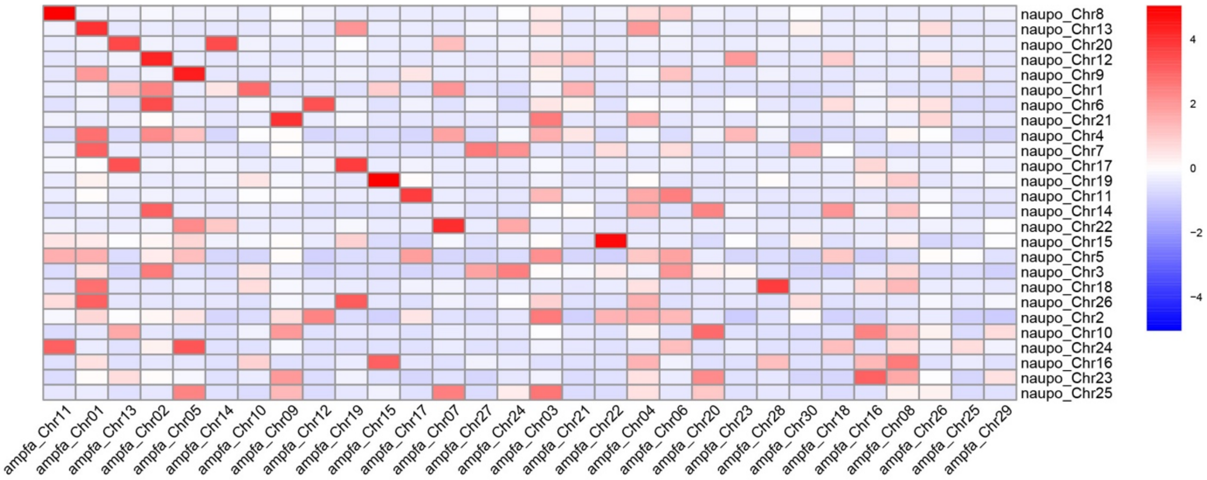

Fig. s5 | Heat map of homologous genes in pairwise chromosomes of *O. sinensis* (octsi_) and *N. pompilius* (naupo_). Each block represents the number of homologous genes.

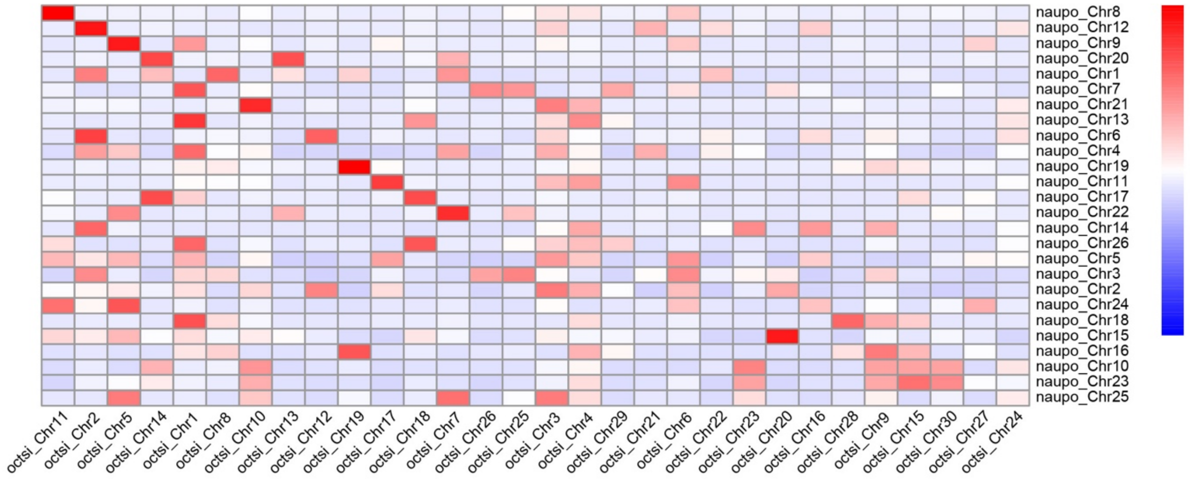

Fig. s6 | Synteny blocks between *A. fangsiao* and *O. sinensis*. **a**) Chromosome synteny analyses among 30 chromosomes of *A. fangsiao* (ampfa) and *O. sinensis* (octsi). **b**) Chromosome synteny analyses between *A. fangsiao* (af_), *O. sinensis* (os_) and *N. pompilius* (np_).

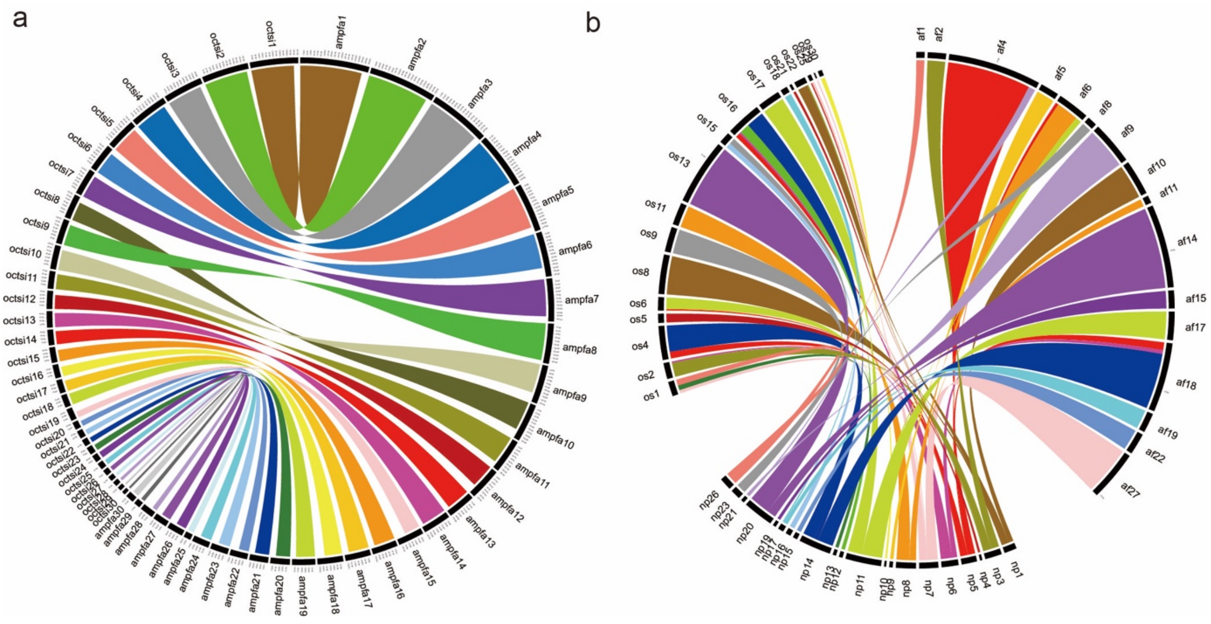

Fig. s7 | (**a**) An overall view of the genomic organization of protocadherin (PCDH), GPCR and C2H2 ZNF in *A. fangsiao* (left) and *O. sinensis* (right). The chromosome relationships are derived from Fig. s6, and are linked by orange arrow at middle. The black arrows at sides point out the chromosomes that contain gene clusters. C2H2 ZNF clusters in the chromosomes of *A. fangsiao* (**b**) and *O. sinensis* (**c**).

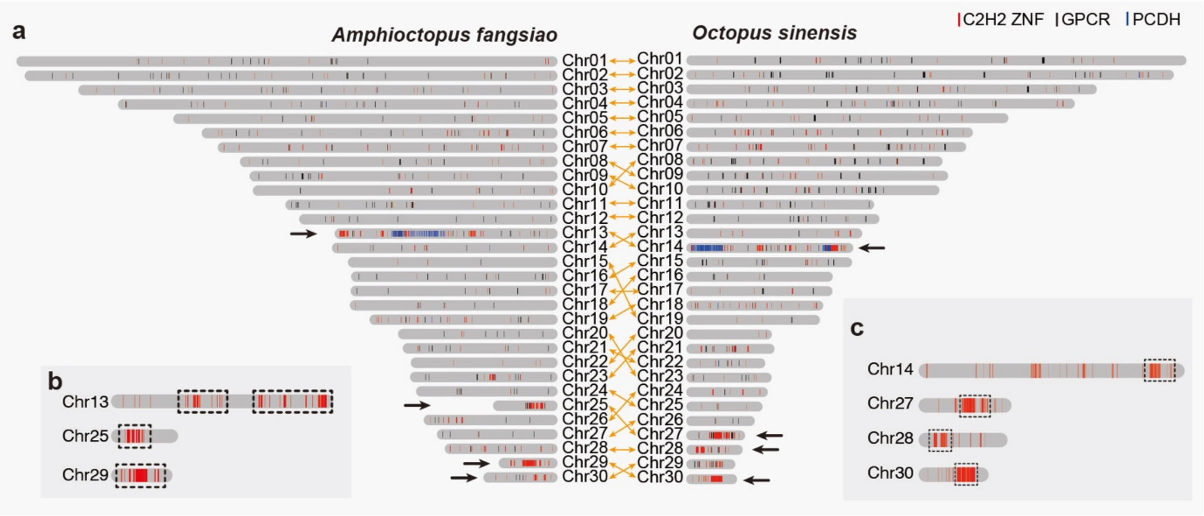

Fig. s8 | Divergent time of C2H2 zinc finger transcription factor (C2H2 ZNF) in *A. fangsiao* (ampfa, top), *O. sinensis* (octsi, middle) and *O. bimaculoides* (octbi, bottom).

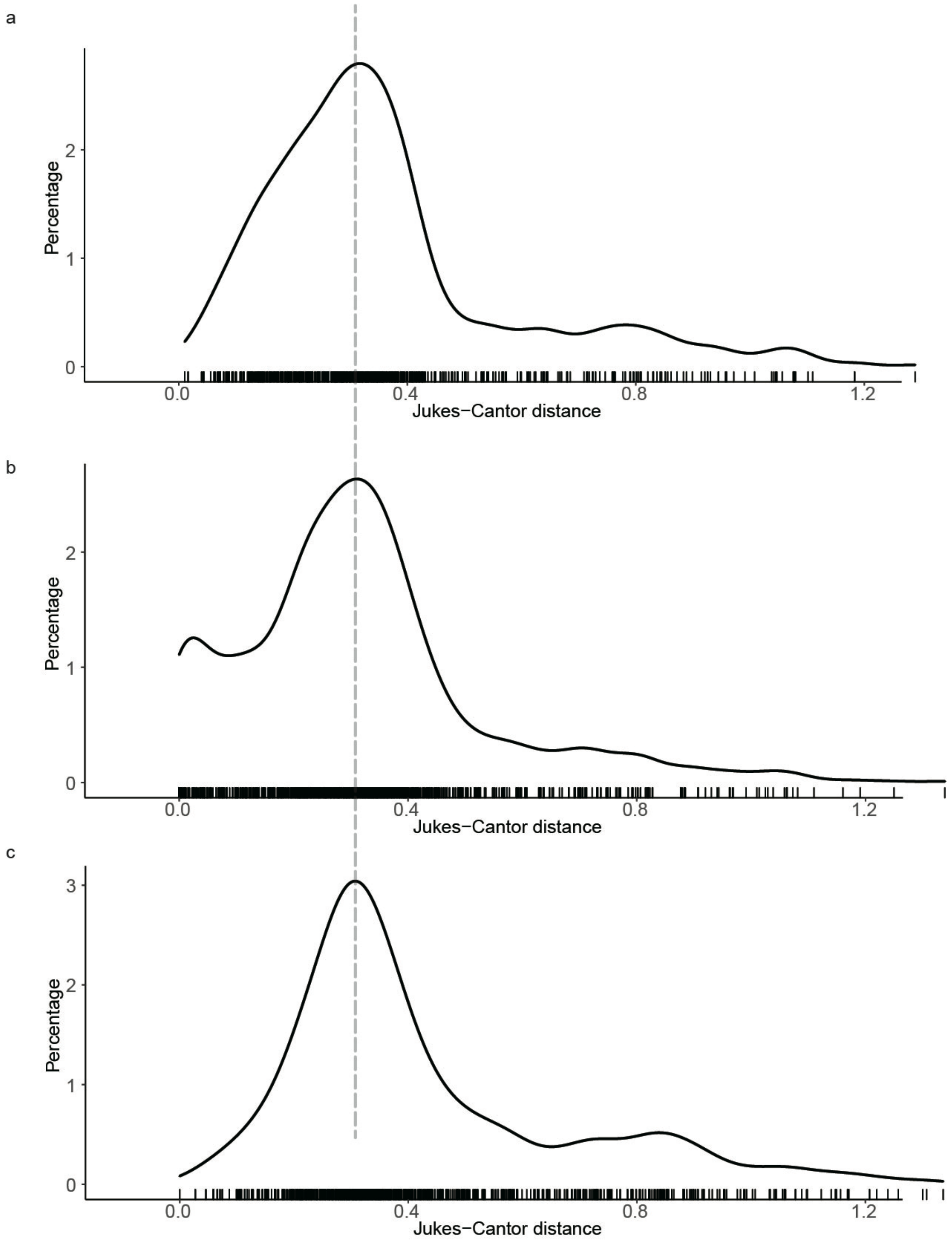

Fig. s9 | Cell population composition of the supra-esophageal brain of *A. fangsiao*. **a**, Comparison of Uniform Manifold Approximation and Projection (UMAP) representation of transcriptomes derived from 10x Genomics (right) and DNBelab C4 (left) platforms (Supplementary data 2). **b**, Percentage of cells that derived from two sequencing platforms in each cluster (Table s9). **c**, Uniform Manifold Approximation and Projection (UMAP) representation of transcriptomes derived from the integration of 10x Genomics and DNBelab C4 platforms.

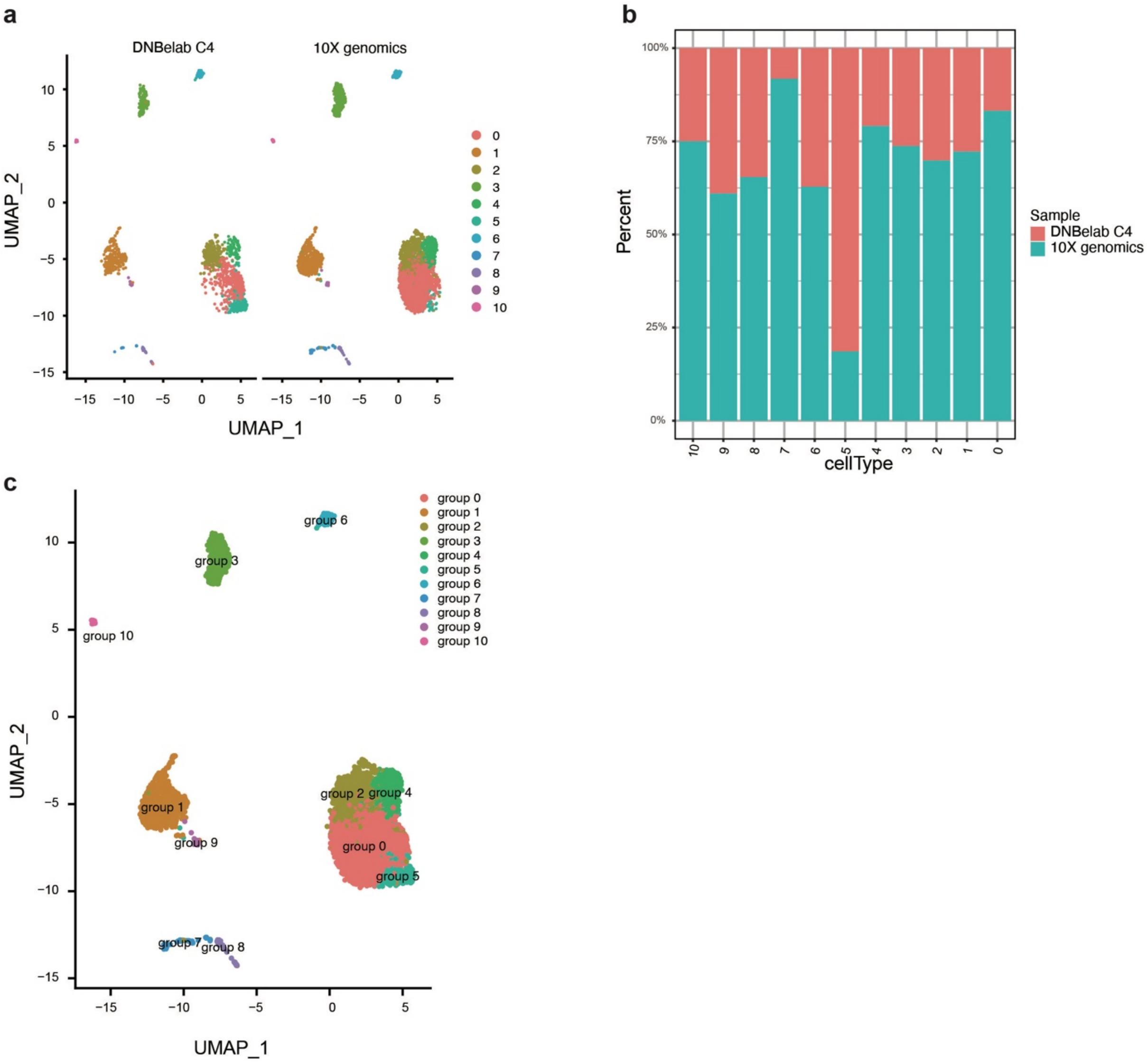

Fig. s10 | Expression of top 10 marker genes of cell type 1-8.

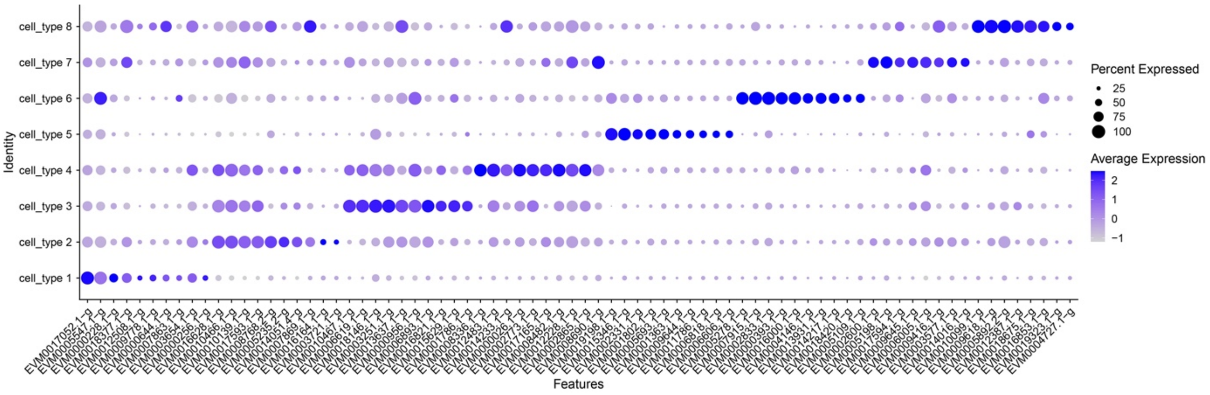

Fig. s11 | Comparisons of functions in cell type 2-8.

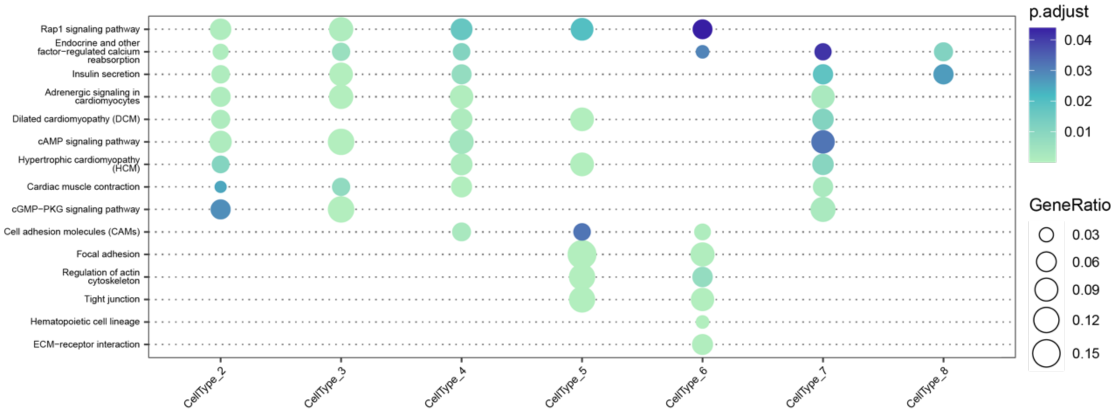

Fig. s12 | Bulk transcriptomic expression of 10 marker genes of cell type 1-8.

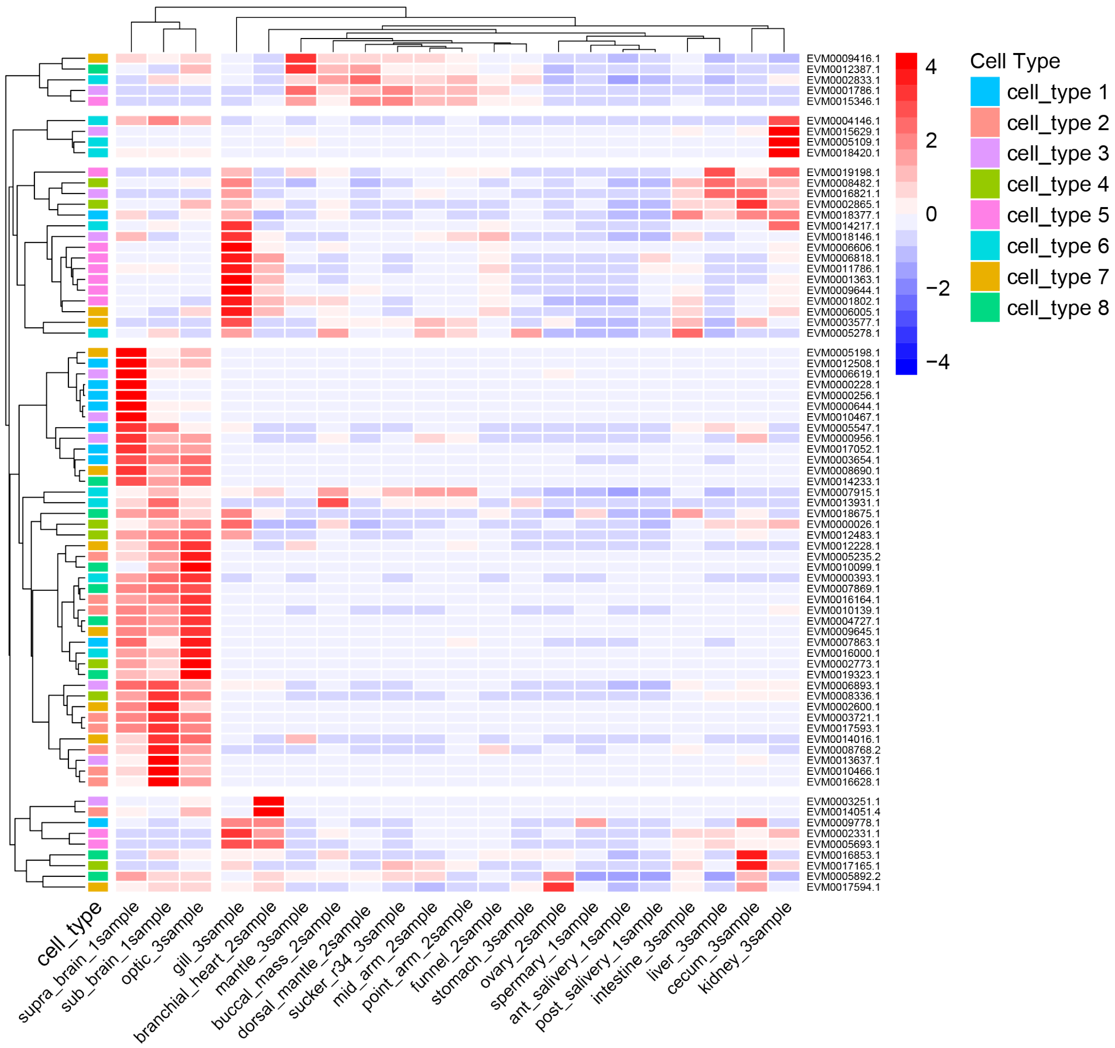

Fig. 13 | Bar plot reflected the number of C2H2 ZNF (top), protocadherin (middle), and GPCR (bottom) genes in marker genes of cell type 1-8.

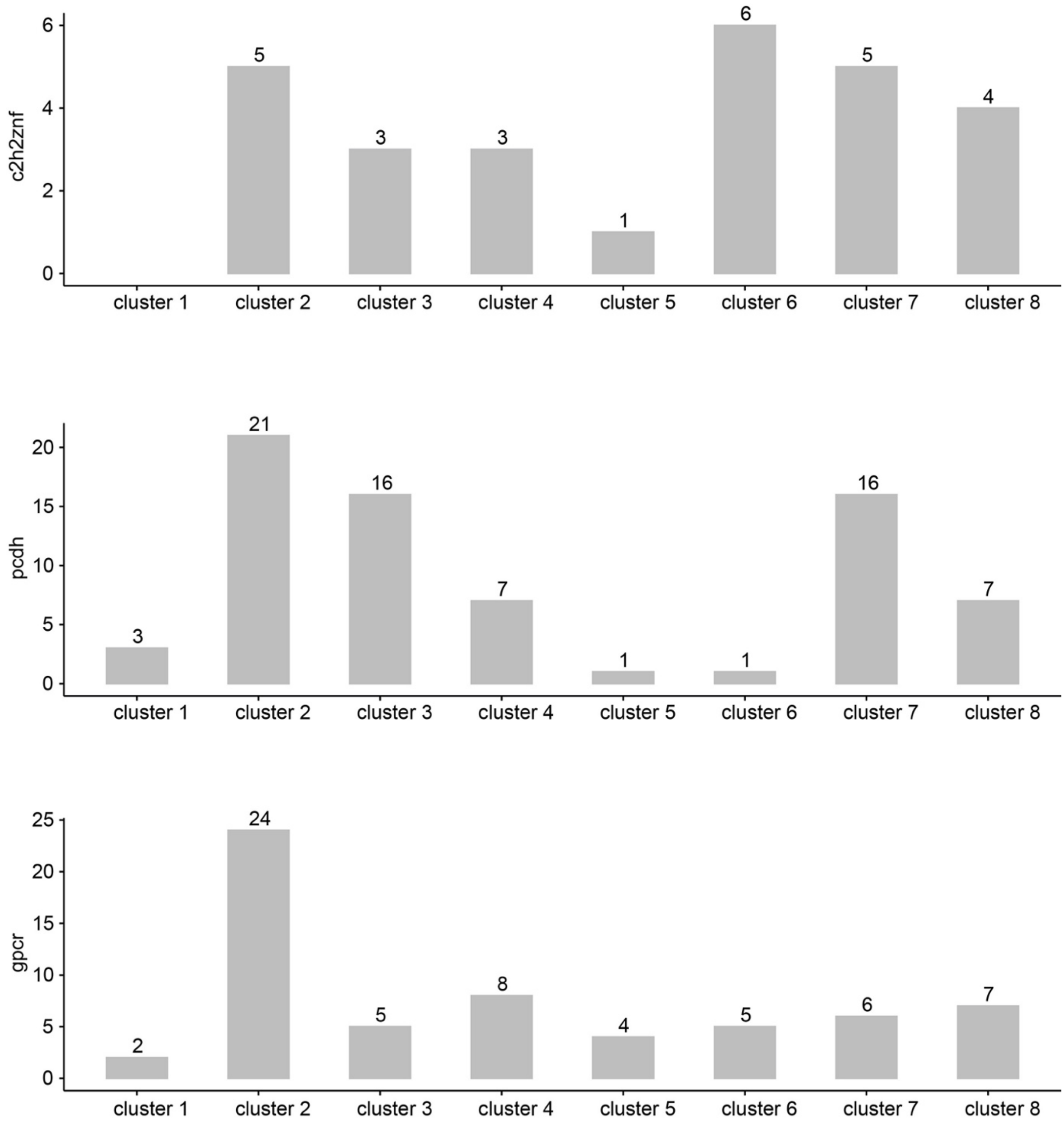

Fig. 14 | Heatmap of GPCR, C2H2 ZNF, and protocadherin (x axis) expressions in the sup-brain cell types (x axis). The GPCR, C2H2 ZNF, and protocadherin genes were the marker genes in each cell type.

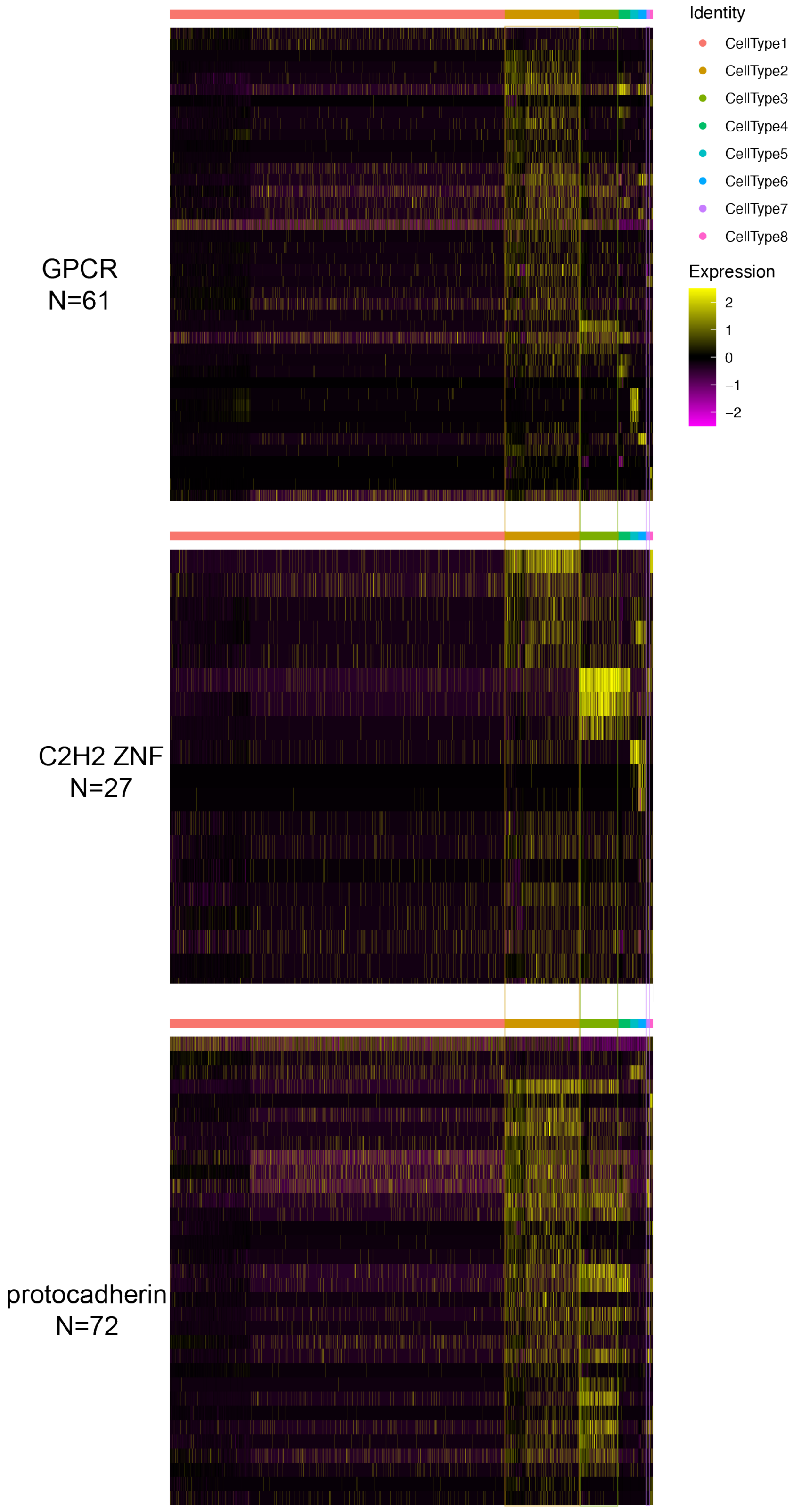

Fig. 15 | Boxplot of average expression of protocadherin (a), GPCR (b), and C2H2 ZNF (c) in cells. The plot was divided by cell types (x axis), and each dot represented the average expression of a protocadherin (GPCR, or C2H2 ZNF) gene in a cell. Paired comparisons between cell type 2 and other cell types were analyzed by a Wilcoxon rank sum test on R v4.0.3 platform, and the *p* values were listed above each box.

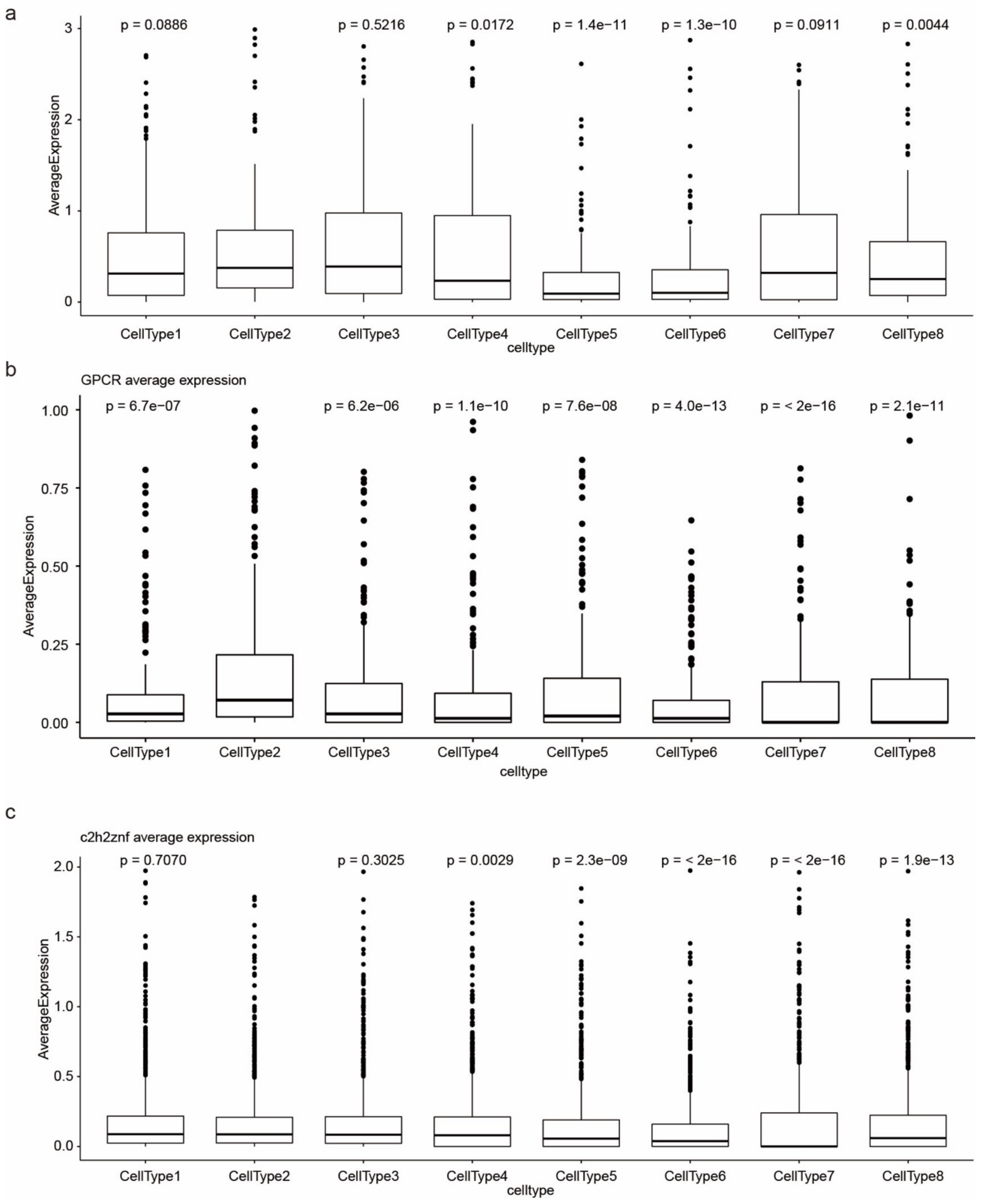

Supplement: Supplementary file 2 — Additional file 2: Fig. s1. The genetic linkage map of Hi-C data. Fig. s2. Phylogenetic analyses based on ML and Bayesian inference methods. Fig. s3-5. Heat map of homologous genes in pairwise chromosomes of A. fangsiao and O. sinensis and N. pompilius. Fig. s6. Synteny analyses between A. fangsiao, O. sinensis, and N. pompilius. Fig. s7. Genomic organization of protocadherin, GPCR and C2H2 ZNF in A. fangsiao and O. sinensis. Fig. s8. Divergent time of C2H2 ZNF. Fig. s9. Cell population composition of the supra-esophageal brain. Fig. s10. Expression of top 10 marker genes of cell type 1-8. Fig. s11. Comparisons of functions in cell type 2-8. Fig. s12. Bulk transcriptomic expression of 10 marker genes. Fig. s13. Bar plot reflected the number of C2H2 ZNF (top), protocadherin (middle), and GPCR (bottom) genes in marker genes of cell type 1-8. Fig. s14. Heatmap of GPCR, C2H2 ZNF, and protocadherin (x axis) expressions in the sup-brain cell types (x axis). Fig. s15. Boxplot of average expression of protocadherin (a), GPCR (b), and C2H2 ZNF (c) in cells. [file 12915_2022_1500_MOESM2_ESM.pdf]
